# Receptor Tyrosine Kinase Signaling and Targeting in Glioblastoma Multiforme

**DOI:** 10.3390/ijms22041831

**Published:** 2021-02-12

**Authors:** Manali Tilak, Jennifer Holborn, Laura A. New, Jasmin Lalonde, Nina Jones

**Affiliations:** Department of Molecular and Cellular Biology, University of Guelph, Guelph, ON N1G 2W1, Canada; mtilak@uoguelph.ca (M.T.); jholborn@uoguelph.ca (J.H.); lnew@uoguelph.ca (L.A.N.); jlalon07@uoguelph.ca (J.L.)

**Keywords:** glioblastoma multiforme, receptor tyrosine kinase, calcium, tyrosine kinase inhibitors

## Abstract

Glioblastoma multiforme (GBM) is amongst the deadliest of human cancers, with a median survival rate of just over one year following diagnosis. Characterized by rapid proliferation and diffuse infiltration into the brain, GBM is notoriously difficult to treat, with tumor cells showing limited response to existing therapies and eventually developing resistance to these interventions. As such, there is intense interest in better understanding the molecular alterations in GBM to guide the development of more efficient targeted therapies. GBM tumors can be classified into several molecular subtypes which have distinct genetic signatures, and they show aberrant activation of numerous signal transduction pathways, particularly those connected to receptor tyrosine kinases (RTKs) which control glioma cell growth, survival, migration, invasion, and angiogenesis. There are also non-canonical modes of RTK signaling found in GBM, which involve G-protein-coupled receptors and calcium channels. This review uses The Cancer Genome Atlas (TCGA) GBM dataset in combination with a data-mining approach to summarize disease characteristics, with a focus on select molecular pathways that drive GBM pathogenesis. We also present a unique genomic survey of RTKs that are frequently altered in GBM subtypes, as well as catalog the GBM disease association scores for all RTKs. Lastly, we discuss current RTK targeted therapies and highlight emerging directions in GBM research.

## 1. Introduction

### 1.1. Gliomas

**Figure 1:**
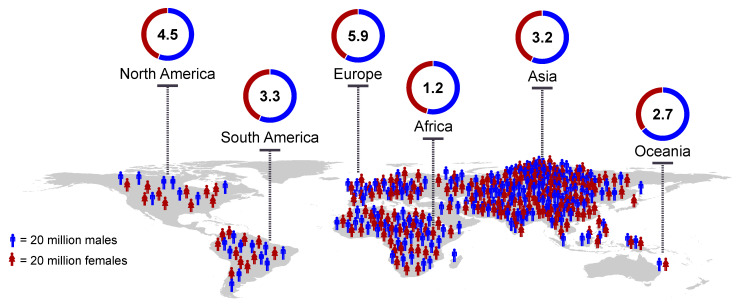
Global CNS tumor incidence rates. Age-adjusted global CNS tumor incidence rates (per 100,000 persons) and the proportions of diagnosed males (blue) and females (red) across all major continents. Number of people represents proportions of males and females in the overall population (downloaded from www.population.un.org, accessed on 10 December 2020). World map and incidence rate charts were plotted using R packages rworldmap and ggplot2, respectively.

Amongst all causes of death worldwide, cancer is in the top ten, with an estimated ten million deaths globally in the year 2018 alone [1]. With regard to malignant neoplasms, those originating in the central nervous system (CNS) are particularly lethal. Approximately 30% of brain and CNS tumors, and over 80% of malignant tumors, are gliomas [2], with glioblastoma multiforme (GBM) being the most frequent and aggressive of all gliomas [3,4,5]. The incidence rate of CNS tumors is higher in populations with European ancestry [1] and shows a predominance in males (Figure 1) [6,7].

**Figure 2:**
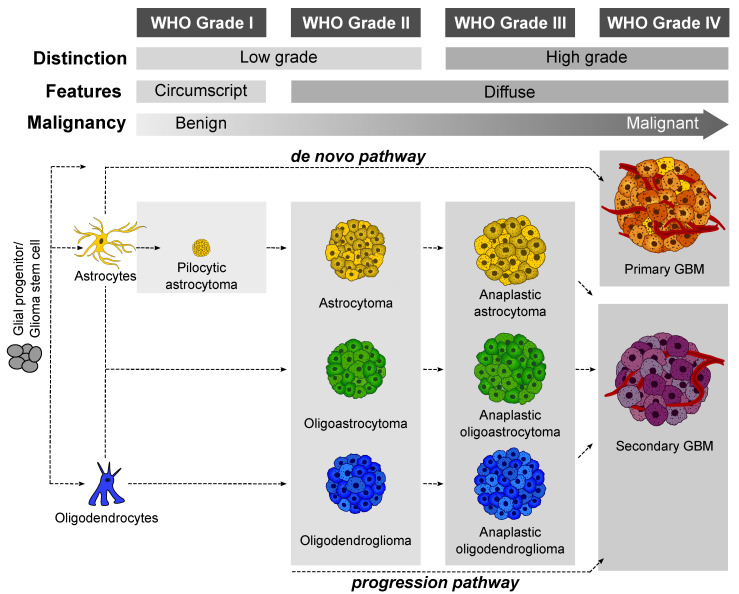
The four glioma grades and their cells of origin as defined by the World Health Organization (WHO, Geneva, Switzerland). Gliomas are characterized on the basis of glial cell types present in the tumor. Tumors arising from astrocytes are called astrocytomas and those originating from oligodendrocytes are called oligodendrogliomas. Tumors consisting of both astrocytes and oligodendrocytes are called oligoastrocytomas. Primary glioblastomas arise mainly from astrocytes and lower-grade gliomas may transform into more aggressive, secondary GBMs.

Gliomas originate from the non-neuronal or glial portion of the brain, namely astrocytes, oligodendroglial, microglial, or ependymal cells [8]. While low-grade gliomas are typically confined to a region, which makes surgical resection possible, higher-grade, diffuse gliomas such as GBM are often infiltrative in nature and near impossible to eliminate [9]. Notwithstanding their initial innocuous façade, some low-grade tumors have the propensity to become more aggressive over time [5,10]. Based on such characteristics as mitotic activity, proliferation, and degree of necrosis, the World Health Organization (WHO, Geneva, Switzerland) classifies gliomas into four distinct grades (Figure 2).

- **Grade I**: Low-grade benign lesions such as pilocytic astrocytomas that exhibit limited proliferation and are frequently seen in children;
- **Grade II**: Low-grade but infiltrative lesions with a tendency to show higher recurrence after surgical resection;
- **Grade III**: Intermediate–high-grade lesions with relatively higher mitotic activity and evidence of malignancy;
- **Grade IV**: High-grade malignant lesions that possess higher mitotic activity, microvascular proliferation, high degree of necrosis, and worst prognosis [3,5]. Grade IV astrocytoma is classified by WHO as GBM [3,5,8].

### 1.2. Glioblastoma Multiforme (GBM)

GBMs are broadly categorized as primary (de novo) and secondary types (Figure 2). The majority of diagnosed cases (90%) are primary, rapidly growing GBMs that lack pre-existing lesions [10]. They are classified as Grade IV tumors at the outset and they occur in older patients (mean age: ~60 years). By contrast, secondary GBMs are more frequently seen in younger cohorts (mean age: <45 years) [3,8,11] and they develop as a result of malignant progression from lower-grade gliomas. For instance, diffuse astrocytomas (Grade II) transform into malignant Grade III/IV gliomas within a decade of initial diagnosis [10,11]. Despite their diverse clinical histories, the two classes of GBM are virtually indistinguishable from each other and show equally poor prognoses.

As implied by the term “multiforme”, GBMs exhibit significant intratumoral heterogeneity. They are characterized by high mitotic activity, microvascular proliferation, genomic aberrations, resistance to apoptosis, necrosis, and invasion into adjacent brain tissue [3,5,8]. Together with cancer stem cell subpopulations driving recurrence as well as varied genetic and epigenetic alterations (discussed below), these features have given notoriety to GBM as one of the most aggressive and difficult-to-treat tumors [3]. The current standard treatment for GBM patients involves neurosurgical resection of the tumor followed by extensive radiotherapy, in combination with the chemotherapeutic agent temozolomide (TMZ), an orally administered alkylating agent that induces DNA lesions in actively proliferating cells [12]. A landmark study by Stupp et al. (2005) examined the efficacy of combining this pharmacological treatment protocol with radiotherapy and reported prolonged overall survival of patients from 12.1 to 14.6 months in addition to a median progression-free survival from 5.0 to 6.9 months when compared to radiotherapy alone [13]. Furthermore, an extended 5-year follow-up study by the same group in 2009 revealed survival for combined therapy to be 9.8% versus 1.9% for radiotherapy alone [14]. This benefit has also been shown by other studies included in a more recent meta-analysis [15]. As long-term survival rates for patients with GBM remain dismal, there is intense interest in using molecular profiling approaches to develop better targeted therapies.

Despite decades of research, long-term survival rates for patients with GBM remain dismal, though limited treatment options do exist. As such, significant efforts are under way to profile the molecular and cellular pathways that are disrupted in GBM to identify targets for novel therapies and ultimately improve the prognosis of this devastating disease.

## 2. Molecular Classification of GBM

### 2.1. Molecular Subtypes

**Figure 3:**
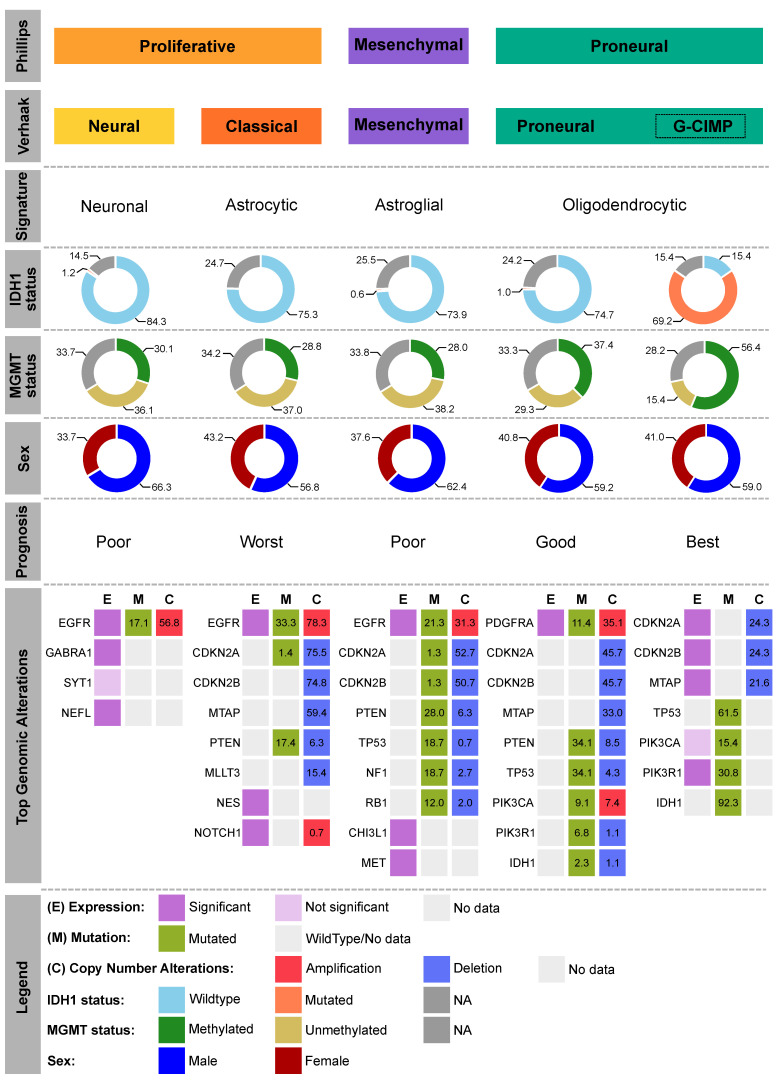
Molecular classification of GBM. GBM tumors in TCGA-GBM dataset originally classified into three (Phillips et al.) [16] or four (Verhaak et al.) [17] distinct subtypes, *CL* (*n* = 146), *ME* (*n* = 157), *NE* (*n* = 83), *PN* (*n* = 99), as well as the glioma-CpG island methylator phenotype (G-CIMP)-positive subset (GC; *n* = 39), with isocitrate dehydrogenase 1 (*IDH1*) mutation status, proportion of males and females, and prognosis shown. Top genomic alterations include highly altered genes, i.e., high mRNA expression (magenta), mutation (green), and/or copy number alterations (red: copy number gain; blue: copy number loss), within each subtype and are sorted according to their overall alteration frequencies. Data were generated by the TCGA Analysis Working Group [20], retrieved from cBioPortal for Cancer Genomics (http://cbioportal.org, accessed on 10 August 2020) [21], and donut charts were plotted using the ggplot2 R package [22].

The lack of clinical advancement with respect to GBM therapy is partly due to limited understanding of its underlying biology. Gene expression profiling studies conducted by various groups during the past two decades have helped to further characterize GBM into several subclasses reflecting survival outcomes [16,17,18]. More specifically, Philips et al. (2006) [17] identified three discrete subclasses of high-grade gliomas: proliferative based on increased tumor-cell Ki-67 labeling index and overexpression of proliferation marker genes *PCNA* and *TOP2A*, as well as mesenchymal and proneural based on increased expression of mesenchymal and proneural marker genes, respectively [17]. A study later conducted by Verhaak et al. (2010) [18] utilized then newly generated data by The Cancer Genome Atlas (TCGA) consortium and identified four molecular subtypes—classical (CL), mesenchymal (ME), proneural (PN), and neural (NE)—on the basis of distinct gene expression signatures of astrocytic, astroglial, oligodendrocytic, and neural origins, respectively (Figure 3), whose characteristics strongly paralleled those observed in the earlier study. The NE subtype has since been proposed to have originated from normal neural tissue at the tumor margin [19].

The CL subtype is strongly associated with amplification and mutation of epidermal growth factor receptor (*EGFR*), along with deletion of cyclin-dependent kinase inhibitors (*CDKN2A/2B*) and gain of chromosome 7, concomitant with loss of chromosome 10, and its overexpression correlates strongly with an astrocytic signature (Figure 3). The ME subtype is associated with mutation and deletion of neurofibromin (*NF1*) and phosphatase and tensin homolog (*PTEN*), as previously concluded [17]. Interestingly, this subtype correlates equally with neural, astrocytic, and oligodendrocytic gene signatures. The PN subtype, featuring an oligodendrocytic signature, is associated with younger age and prolonged survival on account of platelet-derived growth factor receptor α (*PDGFRA*) abnormalities and isocitrate dehydrogenase (*IDH1*) and tumor suppressor *TP53* mutations, all of which are characteristics of secondary GBMs [23]. Lastly, the contested NE subtype is associated with amplification of *EGFR* and deletion of *PTEN* but correlates with neural gene signatures. In addition to EGFR and PDGFRα, several other receptor tyrosine kinases (RTKs) are frequently altered in the various subtypes and will be discussed in more detail below.

Along with genetic and phenotypic differences in GBM subgroups, ongoing refinements in GBM classification have also revealed distinct epigenetic alterations. Of particular importance is the identification of a cluster of GBM tumors characterized by high copy number alterations and altered methylation patterns, referred to as the glioma-CpG island methylator phenotype (G-CIMP) [24]. This subset of tumors is strongly associated with mutations in *IDH* (detailed below) and *TP53* and is more often found in younger patients [24]. Of note, G-CIMP+ tumors are also enriched in the PN subtype and typically portray a favorable outcome [24]. As with *IDH* mutation, the G-CIMP phenotype is also closely associated with methylation of the O^6^-methylguanine-DNA methyltransferase (*MGMT*) promoter, irrespective of glioma grade [25]. MGMT reverses DNA damage induced by alkylating agents such as TMZ. Methylation of the *MGMT* promoter silences the gene, thereby resulting in inefficient DNA repair [26,27]. However, there is evidence that overexpression of MGMT is a determinant in resistance to TMZ therapy [28], which poses a challenge to currently approved GBM therapies. 

### 2.2. Biomarkers

An additional outcome of the TCGA Analysis Working Group examination of 543 cases of GBM [20] is identification of core pathway alterations responsible for glioma occurrence and progression. The following well-known oncogenes and tumor suppressors were found to be significantly mutated in GBM: *EGFR*, *IDH1*, *NF1*, *PDGFRA*, *PIK3R1*, *PIK3CA*, *PTEN*, *RB1*, and *TP53* [20]. Of these genes, the IDH family has recently emerged as an important biomarker. The status of isocitrate dehydrogenase 1 and 2 (*IDH1/2*) enzymes is a major highlight of the recent WHO classification system [11], with *IDH1* now being considered a key reference gene for the classification of GBM tumors. Mutations in *IDH1* appear to preferentially occur in younger glioblastoma patients (mean age: 33 or 41 years) as opposed to wild-type carriers (mean age: 53 or 56 years) and are mostly detected in patients with secondary GBM [8,29]. Curiously, these patients had a longer median survival rate of 3.8 years, compared to 1.1 years for patients with wild-type *IDH1* [8]. Over 90% of *IDH1* mutations reflect an amino acid substitution at an evolutionarily conserved residue (R132H) located within the enzyme’s binding site [30], akin to well-known activating alterations in *BRAF*, *KRAS*, and *PIK3CA* [8,31,32]. Additional studies investigating the functional impact of *IDH1* mutations proposed that IDH1 may function as a tumor suppressor that, when inactivated by a mutation, likely contributes to tumorigenesis [33]. The prognostic role of MGMT has been demonstrated by several studies and therefore serves as an excellent biomarker with respect to its methylation status and expression levels, which can be used independently to predict patient survival and treatment response [34]. In fact, integration of *IDH* mutation and *MGMT* promoter methylation status, together with application of molecular information derived from several “-omics” approaches, may have the potential to substantially improve the classification and prognostication of gliomas. 

Nonetheless, historically, immunohistochemistry (IHC) has dominated the field of cancer classification and diagnostics. The glial fibrillary acidic protein (GFAP) and oligodendrocyte lineage transcription factor 2 (OLIG2) are two such examples of glioma markers routinely assessed by IHC during diagnosis [3]. GFAP is universally expressed in astrocytic and ependymal tumors [35,36], while OLIG2 is both an oligodendroglial and a stem cell marker highly expressed in diffuse gliomas [37,38]. Over the past decade, CD133 and other such stem cell markers as Nestin, Musashi, and sex-determining region y-box 2 (SOX2) have been investigated as potential targets for GBM therapy [39,40,41,42]. Interestingly, IHC can even be used to determine patient IDH1 mutation status using IDH1-specific antibody recognizing the R132H mutation [43,44,45]. 

## 3. Key Cellular Hallmarks of GBM

### 3.1. Uncontrolled Proliferation

One of the many factors responsible for GBM malignancy is the rapid proliferation of GBM cells [46], which can be driven by mutations resulting in uncontrolled activation of signaling pathways such as those downstream of EGFR. Glial cells are known to exhibit a “go or grow” state [47], a phenomenon which highlights the proliferation/invasion dichotomy and ultimately decides a cell’s fate to either proliferate or invade [48]. Tumor progression is largely determined by phenotypic switching between these states, which, in turn, is influenced by microenvironmental changes such as hypoxia, matrix composition, and cytoskeletal dynamics [49,50].

### 3.2. Invasion and Metastasis 

While most gliomas are amenable to neurosurgical resection, it is the cells at the invasive front in GBM that often extend beyond the edge of the tumor, thereby evading removal and subsequently contributing to recurrence and poor prognosis. Although metastasis beyond the CNS is rare, localized spread within the CNS is more common. An important aspect of GBM invasion is the production of a thick extracellular matrix (ECM)—a complex proteinaceous network which occupies the space between neurons and glial cells and comprises roughly 20% of the total brain volume. It facilitates invasion by regulating the activity of proteolytic enzymes. One mechanism by which invasive glioma cells infiltrate into the brain parenchyma is through formation of dynamic actin-rich structures called invadopodia that not only provide GBM cells with the ability to adhere to the ECM but also allow degradation of the surrounding tissue through proteolytic activity [51]. A parallel process promoting invasion involves epithelial–mesenchymal transition (EMT), wherein cells acquire a motile phenotype. In GBM, this is achieved through upregulation of transcription factors such as ZEB1/2, SNAIL, SLUG, and TWIST [52]. It is widely believed that the acquisition of mesenchymal features is associated with stemness and that the co-expression of EMT markers with stem cell markers produces a migratory cancer stem cell [53].

### 3.3. Cellular Heterogeneity

GBMs are often composed of multiple different cell populations arising due to clonal evolution and differences in cell plasticity/stemness [54,55]. This intratumoral heterogeneity is driven by the tumor microenvironment, which is composed of several distinct components, such as the aforementioned ECM, as well as perivascular [56], hypoxic [57], and immune niches [58]. In essence, this complex organization is where glioma stem cells (GSCs) reside and interplay with the ECM to stimulate tumor progression, recurrence, and drug resistance [59,60]. In recent years, GSCs have emerged as promising therapeutic targets given their similarity to neural stem cells in terms of self-renewal, proliferative potential, capacity to differentiate into multiple cell lineages, and the presence of cell surface markers [61,62]. Interestingly, GSCs have an elevated invasive potential compared to non-stem tumor cells [63] and are thought to be more resistant to radiotherapy and chemotherapy than differentiated bulk tumor cells [64]. These subpopulations reflect a variety of cellular states that are dynamic in nature and overlap with cell cycle and neurodevelopmental programs [65]. Indeed, it has been shown that GBM tumors, despite exhibiting a dominant subtype, also contain heterogenous mixtures of cells corresponding to different GBM subtypes and hybrid states (cells that score highly for more than one subtype) which parallel cellular diversity in the developing brain [66]. For instance, cells which scored highly for classical and proneural subtypes exist in progenitor states with enhanced stemness signatures, while cells which scored highly for mesenchymal and neural subtypes exist in differentiated states [66]. In other words, cells conforming to each of the four GBM subtypes portray distinct stemness signatures [67].

### 3.4. Angiogenesis

A notable difference between GBM and lower-grade gliomas is the high degree of microvascular proliferation in GBM. Neovascularization occurs through multiple strategies employed by GBM cells, including vascular co-option and angiogenesis [68]. Angiogenesis drives the formation of new blood vessels from pre-existing ones and generally follows vessel co-option via both hypoxia-dependent and -independent pathways [68]. It involves the secretion of pro-angiogenic factors such as angiopoietin (Ang)-1, which, in concert with vascular endothelial growth factor (VEGF)-A, has been shown to induce angiogenesis in xenograft models [69], and Ang-2, which can mediate tumor angiogenesis in astrocytomas [70]. Transdifferentiation of GSCs into endothelial cells is yet another strategy that enhances neovascularization [71] and VEGF-A has been shown to be secreted by GSCs in extracellular vesicles [72].

The establishment of sufficient and suitable vasculature is necessary for tumor cell growth and proliferation. Normally, vasculature is a neatly organized network of evenly distributed arteries, veins, and capillaries [73]. In GBM (and other cancers), however, the vasculature is disorganized and chaotic, and the lack of pericytes in the periphery results in hyperpermeability or leakiness of blood vessels, leading to edema, which eventually serves as a platform for GBM and endothelial cell migration [74]. The most potent stimulator of endothelial cell migration and proliferation and subsequent angiogenesis is hypoxia. Under hypoxic (1–2% O_2_) conditions, hypoxia inducible factor-1α (HIF-1α) is upregulated and binds to and activates hypoxia response elements (HREs) present on the promoters of genes implicated in angiogenesis, such as VEGF-A [75], which is itself upregulated in tumor as well as stromal cells in GBM [76].

### 3.5. Deregulated Cell Energetics

The nutrient-restricted conditions which stimulate vascular proliferation in GBM are also associated with altered cell energetics. In fact, metabolic complications such as type II diabetes (characterized by hyperglycemia) as well as obesity are independent risk factors for poor survival in GBM [77]. In addition to insulin-signaling-related mechanisms that mediate tumor growth and survival [78], a link between epigenetics and cell metabolism exists and is best highlighted by *IDH1* mutations (see Section 2.2), which likely impact metabolism via altered metabolic flux of α-ketogluterate and NAPDH, thereby impairing normal biosynthetic pathways [79,80]. 

### 3.6. Mutation and Genome Instability

Diverse transcriptional programs also contribute to the extensive variability within GBMs [66]. Tumor development and progression are responsive to genetic (i.e., loss of heterozygosity, mutation, chromosome rearrangement) and epigenetic (i.e., promoter methylation) changes [20]. The most frequently altered genes in GBM encode for signaling proteins which participate in various pathways to maintain the malignant GBM phenotype. Not surprisingly, the predominant mechanism of GBM pathogenicity occurs through dysregulation of cell surface RTKs [81].

## 4. Receptor Tyrosine Kinase (RTK) Pathways Altered in GBM

### 4.1. RTK Regulation and Oncogenic Activation

RTKs are transmembrane proteins comprising a unique extracellular ligand-binding domain, a transmembrane helix, an intracellular tyrosine kinase domain, and a series of tyrosine residues which serve as docking sites for cytoplasmic signaling effectors once phosphorylated [82,83]. In normal cells, receptor activity is tightly controlled and RTK signaling regulates cellular processes such as growth, survival, differentiation, and migration through activation of mitogen-activated protein kinase (Ras/MAPK) and phosphotidylinositol 3-kinase (PI3K/Akt/mTOR) pathways (detailed below in Section 4.2). However, in cancer, RTKs are aberrantly activated and thus contribute to the oncogenic phenotype either by (1) stimulating overproduction of growth factors by cancer cells; (2) overexpression and/or amplification of the RTK itself, enabling hypersensitivity to low ligand concentrations; (3) acquiring mutations in their ligand-binding or kinase domains; (4) fusion of kinase domains with motifs of other, unrelated proteins; or (5) chromosomal translocation, giving rise to a chimeric product with enhanced kinase activity [84,85,86,87,88]. 

**Figure 4:**
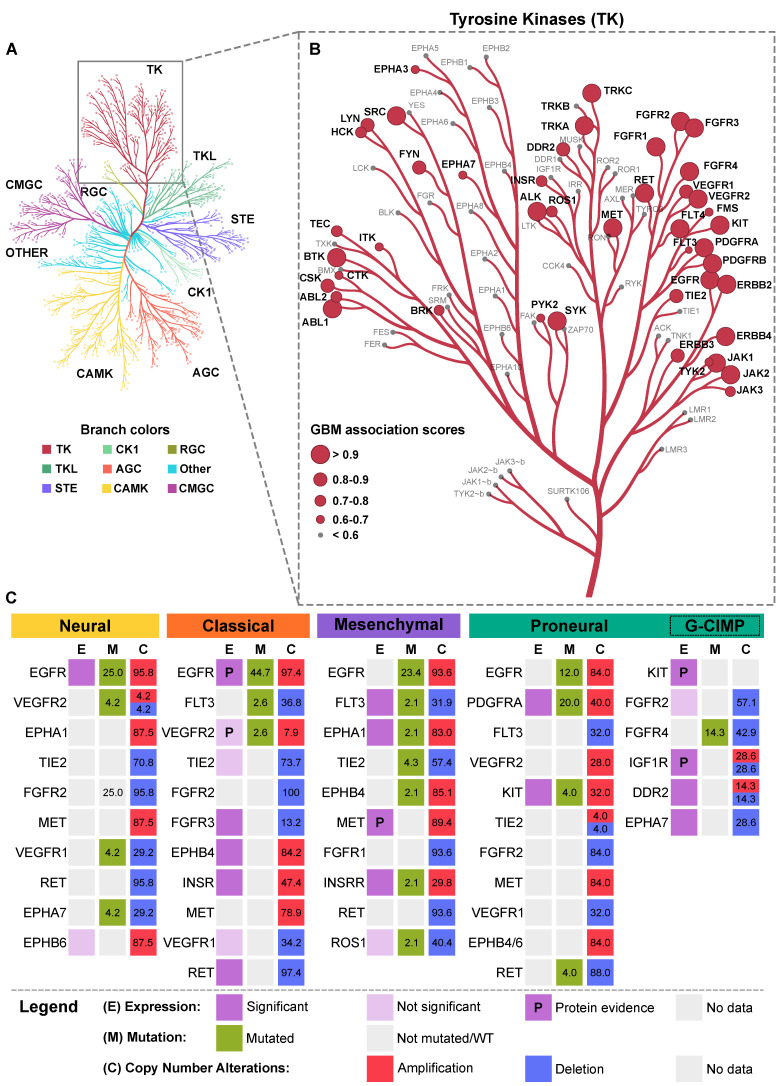
The human kinome and top altered receptor tyrosine kinases (RTKs) in GBM. (**A**) Kinome tree showing human protein kinases grouped on the basis of their sequence similarity. (**B**) Kinases in the tyrosine kinase (TK) group are further classified based on their unique extracellular domain arrangements (not shown). Kinome image, originally conceived by Cell Signaling Technology, Inc. (www.cellsignal.com, accessed on 25 May 2020), based on Manning et al. [91], was accessed and downloaded via the CORAL web application for kinome data visualization [92] and disease association scores for GBM were retrieved from www.targetvalidation.org, accessed on 25 May 2020 [90]. Disease association scores (ranging from 0 to 1) are presented as red circles at the end of each branch, where larger circles represent a higher degree of association. Abbreviations: TK, Tyrosine kinase; TKL, Tyrosine kinase-like; STE, Sterile serine/threonine kinases (STE20, STE11, and STE7-related); CK1, Casein kinases; AGC, Protein A/G/C-related; CAMK, Ca2+/calmodulin-dependent kinases; RGC, Receptor guanylate cyclases; CMGC, CDK/MAPK/GSK-3/CDK-like kinases. (**C**) Subtype-specific RTK alterations in TCGA-GBM cohort. Analysis was performed on complete samples, i.e., only samples with RNA-Seq, mutation, and copy number aberrations (CNA) information (total *n* = 141) were selected for analysis. mRNA expression (magenta) was determined, and mutation (green) and CNA (red: amplification; blue: loss) frequencies were then calculated for all RTKs in each of the four GBM subtypes as well as in the G-CIMP subset (NE, *n* = 24; CL, *n* = 38; ME, *n* = 47; PN, *n* = 25; GC, *n* = 7). RTKs are ordered based on their overall alteration frequencies within the dataset and numbers inside squares indicate percentage of total samples altered. RTKs with supporting protein evidence from Reverse Phase Protein Array are indicated with a “P”. Data were generated by the TCGA Analysis Working Group [20] and retrieved from cBioPortal for Cancer Genomics (http://cbioportal.org, accessed on 10 August 2020) [21].

In humans, 58 RTKs have been identified so far that are categorized into 20 distinct classes based on sequence and structural similarities in their extracellular regions [89]. Of these, a number of RTKs are in the TCGA-GBM dataset. As shown in Figure 4B, we used the Open Targets Platform [90] to highlight disease association scores for RTKs in GBM as well as catalog RTKs that are frequently altered (either in expression, mutation, or copy number changes) in the various subtypes of GBM (Figure 4). RTKs that appear in multiple subtypes or with high association scores are discussed in detail below.

#### 4.1.1. EGF Receptors

The EGFR family is composed of four structurally related transmembrane receptors: EGFR, ErbB2/HER2, ErbB3/HER3, and ErbB4, encoded by (*EGFR*, *ERBB2*, *ERBB3*, *ERBB4*, respectively) [93,94,95,96]. With more than ten ligands and different binding preferences for intracellular adaptors, they generate a complex signaling network and play a pivotal role in developmental processes as well as homeostasis and cancer [97]. EGFR (also known as ErbB1) is a well-characterized RTK in studies pertaining to signal transduction [98] as well as cancer initiation and progression [99]. Dysregulation, particularly that of EGFR, is known to be associated with a variety of human cancers. Specifically, in GBM, EGFR is amplified in approximately 40–50% of tumors and overexpressed in over 60% of patients [23,100], suggesting a causal role. In the TCGA-GBM dataset that we analyzed, overall, 55% of patients presented with EGFR alterations. Specifically, 44.7% of samples representing the CL subtype were mutated, and 23.4% of ME were mutated. EGFR amplification frequencies are the highest in CL (97.4%), NE (95.8%), and ME (93.6%), followed by the PN subtype (84%). EGFR amplification is only minimal in the G-CIMP subset (14.3%). Interestingly, protein expression data from Reverse Phase Protein Array (RPPA) shows that EGFR and its phosphorylated variants (pY992, pY1068, and pY1173) are significantly enriched in the CL subtype (Figure 4). A notable and commonly occurring mutation is the EGFR*vIII* variant, which lacks a portion of the extracellular receptor domain, is activated independent of its ligand, and is constitutively phosphorylated [101]. Interestingly, the variant is only expressed in a subset of GBM tumor cells [102] and co-expression of EGFR*vIII* with EGFR leads to its phosphorylation by the latter and subsequently activates the STAT3/5 pathway, leading to malignant progression [103]. 

ErbB2 is particularly well-characterized in breast cancer, where its expression is considered a prognostic factor [104]. In GBM, elevated expression levels of the receptor have been reported [105,106]. ErbB3 is normally implicated in cell division and survival and has also been reported to be upregulated in some high-grade gliomas [107], whereas ErbB4 is crucial in cardiac muscle differentiation, axon guidance, and neuronal migration during development [108]. Despite its low mRNA expression in GBM, activation of the receptor is associated with increased proliferation, angiogenesis, tumorigenicity, and decreased sensitivity to anti-EGFR therapy [109]. In the TCGA-GBM dataset, while *ERBB2-4* are not mutated in any of the four subtypes, there was notable copy number loss for *ERBB2* in ME samples (23.4%) and *ERBB3* in NE and PN samples (12.5% and 20.0%, respectively), suggesting that these receptors may play a more secondary role in GBM compared to EGFR. RPPA data shows significant enrichment for ErbB2 in the NE subtype, and for its phosphorylated variant pY1248 in the CL subtype, in contrast to phosphorylated ErbB3 protein (pY1298), which is significantly enriched in the PN subtype (data not shown).

#### 4.1.2. Insulin Receptors

The insulin receptor (INSR), INSR-related receptor (INSRR), and insulin-like growth factor (IGF) receptors (IGF1R and IGF2R) play crucial roles in prenatal and postnatal development [110]. INSR, through its two developmentally regulated isoforms, the fetal variant INSR-A and the adult variant INSR-B, regulates key processes such as glucose metabolism and glycogen synthesis [111,112], while IGF1R regulates cell proliferation and differentiation in the developing brain, where it is highly expressed [113]. INSRR works as an extracellular pH sensor—a property defined by its extracellular region, which plays a crucial role in pH homeostasis [114]. In GBM, IGF1R is overexpressed compared to normal tissue [115] and is necessary for neoplastic transformation [113,116]. Moreover, high IGF1R expression is associated with chemoresistance to TMZ as well as reduced survival, suggesting that the receptor may be used as a biomarker [115]. Apart from IGF1R overexpression, IGF1R mutations are rarely observed in GBMs, and in the TCGA-GBM dataset that we analyzed, *IGF1R* is only minimally mutated in the CL subtype (2.5%) but a significant portion of samples show deletion of the gene in the PN subtype (40%). Interestingly, RPPA data shows IGF1R to be significantly enriched in GC, suggesting that the receptor may be strongly regulated. On the other hand, no mutations in *INSR* are detected but the gene is amplified in all subtypes apart from NE. In contrast, *INSRR* is both mutated and amplified (29.8%) in ME, suggesting that the receptors may have subtype-specific roles in GBM. 

#### 4.1.3. PDGF Receptors

The PDGF family of receptors consisting of PDGFRα and PDGFRβ normally play critical roles in embryo development. For instance, PDGFRα is crucial for oligodendrocyte development [117] while PDGFRβ is important for blood vessel formation [118]. Despite structural similarities, PDGFRα and PDGFRβ contribute to GBM development in different capacities. For instance, in the TCGA dataset, PDGFRα is among the most amplified receptors in GBM, second only to EGFR and preferentially upregulated in proneural GBMs, which harbor oligodendrocytic signatures [18]. *PDGFRA* rearrangements such as the *PDGFRA-KDR* gene fusion have also been detected [119]. On the other hand, PDGFRβ is preferentially expressed in glioma stem cells and regulates levels of stem cell markers such as SOX2 [120]. In the TCGA-GBM dataset, *PDGFRA* is highly amplified (40%) and mutated (20%) in the PN subtype, whereas *PDGFRB* is mutated to a small extent in the CL (2.6%) and PN (4.0%) subtypes, with copy number loss (57.1%) prominent in the G-CIMP subset.

The PDGFR family also comprises other well-known receptors such as c-KIT and FLT3, which are implicated in GBM. c-KIT (also known as stem cell factor (SCF) receptor/CD117) is normally expressed in different regions of the CNS during normal brain development as well as endothelial and mast cell progenitors and promotes angiogenesis [121]. In GBM, *KIT* overexpression and amplification has been reported, and amplification as opposed to mutation appears to be the common mechanism underlying its expression [122]. In the TCGA-GBM dataset, *KIT* is mutated in ME (4.3%) and is amplified (32%) and mutated (4%) with significantly elevated mRNA as well as protein expression in the PN subtype. 

Fms-like tyrosine kinase 3 (FLT3) is normally expressed by hematopoietic stem or progenitor cells and plays an important role in the early stages of both myeloid and lymphoid lineage development [123]. In GBM, mRNA expression of *FLT3* and its natural ligand *FLT3L* is detected in astrocytic tumors as well as GBM cell lines [124]; however, the exact mechanism by which FLT3 contributes to GBM occurrence/progression remains unknown. In the TCGA-GBM dataset, *FLT3* has copy number loss in all subtypes (29.2–36.8%), including in the G-CIMP (57.1%) tumor subset, and is mutated in the CL (2.6%) and ME (2.1%) subtypes. 

#### 4.1.4. VEGF Receptors

The VEGF receptor (VEGFR) family contains three RTKs, VEGFR1, VEGFR2, and VEGFR3 (encoded by *FLT1*, *KDR*, and *FLT4*, respectively) [125,126,127], which modulate vasculogenesis and angiogenesis [128], as well as lymphangiogenesis, in the case of VEGFR3 [129]. GBMs express high levels of VEGF and its receptors [130], and the increased angiogenesis and dysfunctional vasculature in these tumors is attributed mostly to aberrant VEGFR2 signaling, which regulates survival, proliferation, migration, and vessel permeability [131]. Furthermore, the hypoxic environment of GBM triggers the “angiogenic switch” [132], wherein the production of HIF-1α induces transcription of VEGF itself [133]. In the TCGA-GBM dataset, VEGFR1 is enriched (but not significantly) in the CL subtype. It is minimally mutated in the ME subtype (2.1%) and deleted in all subtypes, including the G-CIMP subset. VEGFR2 protein expression is significantly upregulated in ME, and the RTK is mutated in the CL (2.6%) and NE (4.2%) subtypes and deleted in PN (28%) as well as the G-CIMP subset (28.6%), while *VEGFR3* is not mutated but a considerable proportion of samples have the gene deleted in the G-CIMP subset (42.9%). 

#### 4.1.5. FGF Receptors

The fibroblast growth factor receptor (FGFR) family consists of four receptors (FGFR1–4) which activate signaling cascades via an intermediary FGFR substrate 2 (FRS2) [134]. FGFR signaling is crucial for normal CNS development [135] as well as for adult neuron and astrocyte survival [136]. Some FGFR aberrations commonly found in GBM are *FGFR1* (and *FGFR3*) translocations that create fusion genes with transforming acidic coiled-coil genes (*FGFR-TACC*), leading to constitutive receptor activation and aberrant nuclear localization/aneuploidy [137]. *FGFR3* mRNA is significantly elevated in the classical subtype, and a recent single-cell RNA-Seq study revealed its expression to be five times higher in infiltrating GBM cells at the migrating front compared to the tumor core [138]. FGFR2, on the other hand, is expressed in glial cells, where its expression is inversely correlated with increasing tumor grade [114]. In the TCGA-GBM dataset, *FGFR1* and *FGFR2* are not mutated at all but *FGFR3* is mutated in ME (4.3%) and *FGFR4* is highly mutated in the G-CIMP subset (14.3%). In terms of copy number alterations, *FGFR2* is deleted in all subtypes—100% of patients representing the CL subtype have this gene deleted and 93.6% of ME samples have this gene deleted. The other three receptors are deleted in G-CIMP and other subtypes but in a very small proportion of samples.

#### 4.1.6. Neurotrophin Receptors

The neurotrophic tropomyosin receptor kinase (*NTRK*) genes (*NTRK1*, *NTRK2*, and *NTRK3*) encode for three tyrosine receptor kinases (TRKs), TrkA, TrkB, and TrkC, that play central roles in mammalian nervous system development and control of synaptic plasticity [139,140]. Through their diverse and variable binding affinities to neurotrophin ligands [141], Trk receptors generate distinct biological responses. For instance, TrkA binds to nerve growth factor (NGF) with incredibly high affinity [142], and it activates proliferative pathways in GBM cells [143]. TrkB, via its activating ligand brain-derived neurotrophic factor (BDNF), plays a pivotal role in neuronal survival [144,145], and increasing evidence also supports a role for TrkB signaling in glioma cell growth [146,147]. Intriguingly, there is a strong link between Trk receptor fusions and gliomagenesis in vivo, particularly in pediatric high-grade gliomas, where Trk receptor fusions are reported in diffuse intrinsic pontine gliomas (DIPGs) and non-brainstem tumors [148]. In the TCGA-GBM dataset, none of the three receptors are mutated, though *NTRK1* is amplified in ME (29.8%). *NTRK2* has comparable levels of copy number amplification and loss across all subtypes, whereas *NTRK3* has notable copy number loss (40%) in PN. 

#### 4.1.7. MET (HGF) Receptor

MET (also known as MNNG-HOS transforming gene/hepatocyte growth factor/HGF/c-MET) receptor is a well-characterized proto-oncogene stimulated by its ligand, the hepatocyte growth factor (HGF) [149]. During embryonic development, MET is important for skeletal muscle growth [150] and epithelial remodeling [149]. In GBM, MET is not only involved in proliferation, migration, invasion, and angiogenesis [151], but also maintenance of stemness [152]. In the TCGA-GBM cohort, *MET* is found to be amplified, with elevated mRNA levels in the ME subtype [23] and copy number alterations in all other subtypes. A notable feature of this receptor is its ability to colocalize with the cytoskeletal protein cortactin and induce its phosphorylation in the absence of Src to facilitate invadopodia dynamics [153]. Dysregulation of MET is associated with increased invasion and poor prognosis [154], making it an excellent prognostic indicator [155]. In the TCGA-GBM dataset, although no *MET* mutations are found, over 80% of samples within each subtype show amplification of the gene and 42.9% of samples in the G-CIMP subset have amplified *MET*.

#### 4.1.8. Eph Receptors

The large erythropoietin-producing human hepatocellular (Eph) receptor family comprises 14 members that are categorized into two structural subclasses, EphA1-8 and EphB1-6 [156]. They associate with membrane-bound ephrin ligands to induce bi-directional signaling [157] and regulate neuronal and neuronal–glial communication [158]. During development, Eph receptors play critical roles in axon guidance and migration of neural crest cells [159,160]. In contrast to normal brain tissue, GBM cells express elevated levels of EphA2 and EphA3 [161]. EphA2 expression is highly correlated with the tumor-propagating potential of GBM cells with stem-like properties [162] while elevated levels of EphA3 help to maintain tumorigenicity in tumor-initiating cells [163]. For other Eph receptors, their altered expression can be observed in all GBM subtypes, and expression of select Eph receptors parallels poor survival in GBM patients [164]. In the TCGA-GBM dataset, *EPHA1*, *EPHB4*, and *EPHB6* are mutated in the ME subtype (all 2.1%) and amplified in all subtypes, whereas *EPHA7* is mutated in the NE subtype (4.2%) and deleted in all subtypes. Most of the other Eph receptors are not mutated and are mostly deleted in the G-CIMP subset (range of alteration frequencies: 28.6–42.9%).

#### 4.1.9. Tie Receptors

The Tie receptor family consists of two members, Tie1 and Tie2 (encoded by *TIE1* and *TEK* respectively), which are intricately regulated by angiopoietin ligands Ang-1 and Ang-2. Originally discovered in endothelial cells [165,166] and initially thought to be found almost exclusively at the surfaces of endothelial and hematopoietic cells, Tie2 has recently received attention for its presence in the nonvascular compartments of gliomas, and its expression in neoplastic glial cells coincides with glioma progression from lower to higher grades [167,168]. Considerable evidence generated over the last two decades suggests that Tie2 signaling may regulate crosstalk between glioma cells and vascular endothelial cells of the tumor microenvironment [168]. Ang-1 and Ang-2 also impact glioma occurrence. For instance, Ang-2, which functions as a context-dependent Tie2 agonist [169,170], is overexpressed in gliomas, where it can promote invasion through activation of matrix metalloprotease 2 (MMP2) [171,172]. Moreover, it has also been shown to act as a chemoattractant for Tie2-expressing monocytes/macrophages (TEMs), which are a subset of pro-angiogenic cells appearing in response to anti-angiogenesis therapy [173]. These cells are mostly found at the tumor periphery and are associated with an invasive phenotype in gliomas [174,175]. In the TCGA-GBM dataset, Tie2 alterations primarily manifest as copy number losses in the NE, CL, and ME subtypes, the gene is upregulated (but not significantly) in CL and mutated in ME (4.3%), and it is deleted in all subtypes, including the G-CIMP subset. *TIE1* is not mutated in any subtype but is deleted in the G-CIMP subset (42.9%).

#### 4.1.10. DDR Receptors

The discoidin domain receptors (DDR1 and DDR2) bind collagen in the surrounding matrix rather than classical ligands [176] and they exhibit delayed/sustained signaling in contrast to other RTKs [177]. DDRs normally function to orchestrate important cell behaviors, such as proliferation, migration, adhesion, and ECM remodeling [178]. In glioma, collagen levels are elevated, leading to changes in DDR signaling, as well as alterations in ECM stiffness, which may further influence tumor progression [179]. DDR1 is highly expressed in pediatric high-grade gliomas [180] as well as adult gliomas, irrespective of grades [181], where cells overexpressing the receptor show enhanced invasion and migration, likely as a result of activation of MMP2 [182]. Upregulation of DDR1 has also been shown to be associated with poor clinical outcomes [183]. Only one case of *DDR2*-mutated GBM has been reported thus far and the authors speculate that the mutation contributes to invasion by enhancing tumor cell–ECM interactions [184]. In the TCGA-GBM dataset, while neither receptor is found to be mutated, *DDR2* is significantly elevated in G-CIMP subset and amplified in ME (29.8%), with comparable frequencies of copy number gain and loss observed in other subtypes. 

#### 4.1.11. RET Receptor

The RET oncogene is stimulated by GDNF-family ligands [185]. It is highly expressed during early embryogenesis [186] and plays an important role as a guidance co-receptor during axon growth [187] and neuronal survival [188]. Dysregulation of RET contributes to several forms of cancer, including gliomas, where it has been shown to be highly expressed [189]. Specifically, in GBM, however, *RET* is not considered a mutational driver owing to lower mutation rates [190] as observed in the TCGA-GBM dataset, where *RET* is only slightly mutated in the PN subtype (4%). However, *RET* is significantly elevated in the CL subtype, and a significant portion of samples (upwards of 80%) of all subtypes show copy number loss and 57.1% of G-CIMP samples show deletion of the gene.

#### 4.1.12. ROS Receptors

The *ROS1* gene encodes an orphan RTK that has been characterized as a proto-oncogene [191]. During embryonic development, its expression profile is typically restricted to epithelial cells, where it plays a role in their differentiation [192]. In normal brain tissues, ROS1 is expressed at low levels. However, it is highly expressed in some GBM cell lines [191,193] as well as in surgical GBM samples, where its expression parallels increasing tumor grade and is associated with hypomethylation of a CpG island in the promoter region [194]. This differential expression in glioma grades suggests a role in tumor initiation as opposed to progression. In the TCGA-GBM dataset, *ROS1* is only slightly mutated in the ME subtype (2.1%) and deleted in all subtypes, including the G-CIMP subset. 

### 4.2. RTK Downstream Signaling Pathways

**Figure 5:**
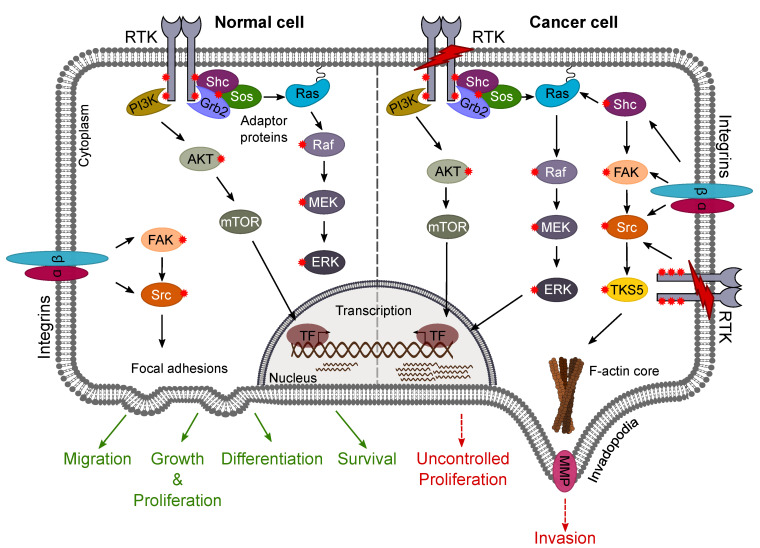
Simplified schematic illustration of common RTK signaling pathways in normal and cancer cells. In normal cells, ligand binding (or integrin-mediated adhesion signals) induces receptor dimerization and tyrosine phosphorylation (red asterisks). Activated RTKs in turn initiate signaling cascades such as the RAS/MAPK, PI3K/Akt/mTOR, or FAK/Src pathways through recruitment of adaptor proteins Grb2 and Shc. This results in cellular responses such as survival, growth, proliferation, differentiation, and migration (left; depicted in green). In cancer cells, overexpression, mutation, or amplification of RTKs can lead to an oncogenic phenotype by activation of invasion pathways as well as uncontrolled proliferation (right; shown in red).

RTK signal propagation requires interaction with intracellular signaling components such as adaptor proteins that function primarily to bridge RTKs and downstream effector proteins (Figure 5). Adaptor proteins inducibly bind to the phosphorylated receptor to activate enzymes such as kinases and GTPases that, in turn, phosphorylate and activate other kinases in well-characterized signaling pathways. RTK signaling ultimately affects such cellular functions as proliferation, survival, and differentiation by altering the expression of target genes in the nucleus [195,196]. Oncogenic changes in RTK signaling lead to uncontrolled cell proliferation and invasion. Three of the most prominently altered RTK pathways in gliomas include the Ras/MAPK, PI3K/Akt/PTEN, and FAK/Src pathways, all of which rely on adaptor proteins such as Grb2 and Shc to relay the signals from the cell membrane.

#### 4.2.1. Ras/MAPK Pathway

The Ras/MAPK pathway is frequently dysregulated in GBM. Ras functions as a guanosine-binding protein (G protein) that cycles between inactive (bound to GDP) and active (bound to GTP) states [197]. Activated Ras (Ras-GTP) promotes GBM progression by mediating cell cycle progression, survival, and migration of GBM cells. Despite increased Ras activity, Ras mutations are rarely encountered in GBM and increased pathway activation may be attributed to other factors such as hyperactivation of RTKs [198].

#### 4.2.2. PI3K/Akt/PTEN Pathway

The PI3K/Akt pathway is a major cell signaling pathway that regulates cell proliferation, survival, growth, and glucose metabolism, amongst other processes [199]. Under normal conditions, the phosphatidylinositol 3-kinase (PI3K)/Akt/mTOR pathway is activated by survival factors through RTKs such as EGFR and Tie2 [200,201]. However, in GBM, the pathway is constitutively active due to PTEN alterations, mutations within the gene encoding the p110 catalytic subunit of PI3K (*PIK3CA*), or Akt amplification [23,202]. Inhibition of the PI3K pathway, therefore, is a promising intervention point for GBM. Furthermore, several studies indicate that there is crosstalk between PI3K and Ras/MAPK pathways [203,204,205], leading to activation of the latter.

Signaling through PI3K/Akt leads to activation of mammalian target of rapamycin (mTOR), an important serine–threonine kinase that forms two distinct multiprotein complexes and modulates cellular processes such as growth response (through mTORC1) [206] and cytoskeletal reorganization (through mTORC2 and Akt) [207]. Since these processes are highly altered in GBM, the PI3K/Akt/mTOR pathway plays a key role in gliomagenesis and progression [208]. 

#### 4.2.3. FAK/Src Pathway

Numerous studies centering on complex interactions of glioma cells and the microenvironment have highlighted the role of two key mediators, focal adhesion kinase (FAK) [209] and the original oncogene Src [210,211], both of which participate in signaling pathways triggered by integrins or indirectly by RTKs. Of note, overexpression of FAK and the parallel increase in its autophosphorylation make it an active therapeutic target [212]. Inhibitors such as Y15, which functions to inhibit FAK autophosphorylation at Y397, have been shown to successfully reduce GBM cell growth, especially in combination with TMZ [213].

#### 4.2.4. Shc Adaptor Proteins

Adaptor proteins known to participate in the above-described signaling cascades include Grb2 and members of the Shc family (ShcA-D). These adaptors are recruited to EGF, Trk, as well as Tie2 receptors [214,215,216,217] and are upregulated in gliomas. Notably, ShcD is unique in its ability to further activate RTKs, presumably through blocking phosphatase-mediated dephosphorylation of the cytoplasmic tail [218,219]. For instance, ShcD can bind and promote ligand-independent activation of EGFR, and its expression parallels EGFR hyperphosphorylation in malignant gliomas [220]. ShcD also binds and induces hyperphosphorylation of Tie2 in glioma cells, and it regulates invadopodia formation, FAK signaling, and invasion [221]. ShcD therefore serves as a novel signaling molecule of interest in glioma.

## 5. Non-Canonical Modes of RTK Activation in GBM

As presented above, dysregulation of RTK-dependent Ras/MAPK, PI3K/Akt/PTEN, and FAK/Src signaling pathways promotes GBM malignancy. However, accumulating evidence suggests that other molecular events connected to RTKs can also influence the migration, proliferation, treatment resistance, and/or survival of these cancer cells. Further understanding of these phenomena could help us to identify novel targets for treatment intervention.

### 5.1. G-Protein-Coupled Receptor-RTK Signaling

G-protein-coupled receptors (GPCRs) represent a diverse group of cell surface receptors that mediate the transduction of extracellular signals to a variety of intracellular pathways. Each GPCR has a related heterotrimeric G protein, which comprises three subunits: Gα, Gβ, and Gγ. Ligand-binding activates GPCRs and causes dissociation of the Gα and Gβγ subunits followed by modulation of downstream effectors [222]. A seminal study 25 years ago made the discovery that application of the GPCR agonists endothelin-1, lysophosphatidic acid, or thrombin to cultured Rat-1 fibroblast cells results in EGFR activation and downstream phosphorylation of MAPK [223]. This finding helped to elucidate non-canonical interactions between GPCRs and RTKs that can be as influential as those following canonical receptor activation (reviewed in [224]). There are two modes of RTK transactivation by GPCRs. In ligand-dependent transactivation, membrane-bound RTK pro-ligands are released by MMPs as a result of GPCR signaling [225,226], resulting in both autocrine and paracrine RTK activation. In ligand-independent transactivation, heteroreceptor complexes are formed between GPCRs and RTKs [227], or GPCR second messengers can interact with the intracellular domain of RTKs [228,229]. Ultimately, both scenarios cause tyrosine phosphorylation of unliganded RTKs that can subsequently stimulate pro-oncogenic cellular processes such as cell proliferation and migration. Notably, evidence of RTK transactivation by GPCR has been collected for different glioma cell types. For instance, μ-opioid receptor activity stimulates ERK signaling downstream of FGFR1 in rat C6 glioma cells [230]. Other examples include enhanced chemotaxis and proliferation of human U87 GBM cells resulting from EGFR signaling mediated by formylpeptide receptor (FPR) activation [231], and increased migration due to GPCR CXC chemokine receptor 4 (CXCR4) activation of PDGFRβ in human GL15 GBM cells [232]. Interestingly, RTKs can inversely transactivate GPCRs by ligand-dependent or ligand-independent mechanisms under certain circumstances [233]; however, evidence of this specific interplay remains to be found in GBM cells.

### 5.2. Calcium Signaling

Calcium (Ca^2+^) is a second messenger that plays a pivotal role in almost every aspect of cellular function [234]. In glioma, Ca^2+^ signaling may even be linked to metastasis and treatment outcomes. Details about these relationships have been extensively reviewed in recent years [235,236]. Here, we focus on two points hinting at relationships between Ca^2+^ homeostasis and specific RTK activity in GBM.

#### 5.2.1. Inositol 1,4,5-trisphosphate (IP_3_) Receptor-Mediated Calcium Signaling

**Figure 6:**
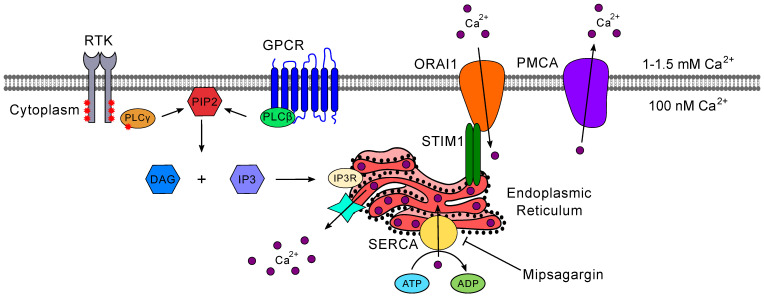
G-protein-coupled receptor (GPCR) and RTK regulation of phospholipase C (PLC) and calcium signaling. GPCRs activate PLCβ while RTKs phosphorylate and activate PLCγ, and these subunits work together to cleave phosphatidylinositol 4,5-bisphosphate (PIP2), found in the cell membrane, into diacylglycerol (DAG) and inositol 1,4,5-trisphospate (IP3). IP3 receptors (IP3R) line the endoplasmic reticulum (ER) membrane, and IP3 binding opens calcium ion (Ca^2+^) channels to allow Ca^2+^ to flow into the cytoplasm. Sarcoendoplasmic reticular Ca^2+^ ATPase (SERCA) transporters maintain stored calcium in the ER. When ER calcium is depleted, stromal interaction molecule (STIM) proteins form multimers that translocate to ER–plasma membrane junctions and recruit Orai channels to initiate calcium entry into the cell. Subsequently, homeostasis is maintained by plasma membrane Ca^2+^ ATPase (PMCA), which transfers Ca^2+^ from the cytosol out of the cell to maintain its gradient against the extracellular environment (~100 nM vs. ~1–1.5 mM, respectively).

In recent years, several studies have established that targeting IP_3_R-derived Ca^2+^ signaling or related pathways downstream of RTK signaling can achieve desirable outcomes against GBM (Figure 6). For example, inhibition of IP_3_Rs with caffeine not only impairs the migration and invasion of GBM cells but also promotes the survival of xenografted mice [237]. An independent study found that trifluoperazine (TFP), an FDA-approved typical antipsychotic drug used to treat schizophrenia, also limits GBM motility and tumor growth by affecting IP_3_Rs [238]. TFP produces disinhibition of the IP_3_R channels indirectly through interaction with the Ca^2+^-binding calmodulin subtype-2 to irreversibly deplete the endoplasmic reticulum (ER) of its Ca^2+^ content. One particular implication of this finding is that disrupting the mobilization of Ca^2+^ stored in the ER appears to be a central factor affecting GBM. This scenario is consistent with promising preclinical results found with mipsagargin (also known as G-202) against solid tumors [239]. Mipsagargin is a SERCA pump inhibitor whose therapeutic effect is attributed to the depletion of ER Ca^2+^ levels due to reuptake failure (Figure 6). Of note, mipsagargin has been successfully shown to promote disease stabilization in patients with advanced hepatocellular carcinoma [240], and a Phase II clinical trial for recurrent or progressive GBM (NCT02067156) was completed in 2017, with results awaiting publication.

#### 5.2.2. Store-Operated Calcium Entry 

Sustained depletion of Ca^2+^ stored in the ER promotes the activity of a mechanism called store-operated Ca^2+^ entry (SOCE), which is the primary route by which extracellular calcium can enter non-excitable cells to control processes such as store refilling, exocytosis, enzyme function, cell proliferation, and gene expression (Figure 6) [241]. With a better understanding of the molecular underpinnings of SOCE, there has been increased interest in the contribution of this pathway to cancer biology. Efforts to date have helped to uncover several connections between dysregulation of SOCE and different types of cancer [242,243]. 

In GBM, increased Orai1 protein levels have been detected alongside higher SOCE activity in patient-derived primary tumor cells [244] and immortalized cell lines [245], while bioinformatic analyses conducted with deposited transcriptomic datasets found evidence of higher expression of *ORAI2* in GBM samples compared to both lower-grade gliomas and normal brain samples [246]. Furthermore, pharmacological inhibition of SOCE with the small molecule SKF96365 or *ORAI1* downregulation by RNA interference were both linked to decreased migration and invasion in GBM cells [156,158]. The reduction in SOCE activity was also found to prevent phosphorylation of the proline-rich tyrosine kinase 2 (PYK2) [245], a FAK family kinase that plays a role in focal adhesion turnover and epithelial-to-mesenchymal transition of glioma cells [247]. While SOCE interplay with RTK activity has not been examined in GBM, work performed with a variety of other cancer cell lines offered the tantalizing result that disruption of EGFR/ErbB2 signaling produces an important decrease in SOCE signal amplitude—a finding suggesting that the anticancer effects of RTK inhibitors may be mediated by inhibition of SOCE [248]. Overall, these studies support the need for further examination of SOCE in GBM biology and an in-depth, systematic evaluation of targeting this pathway to attack these tumor cells.

## 6. RTK Inhibitors and Antagonists with Anti-GBM Properties

GBM treatment is difficult because not only are multiple steps involved in malignant transformation, but the blood–brain barrier presents a challenge in restricting the delivery of crucial chemotherapeutic agents [249]. Given the critical roles of RTK-mediated signaling in glioma occurrence and progression, tyrosine kinase inhibitors (TKIs) have garnered prime interest in recent clinical investigations because of their ability to target one or several of these pathways. Some select molecules and respective antagonists that have attracted the most interest are highlighted below.

### 6.1. EGFR: Erlotinib, Gefitinib, and Afatinib; EGFRvIII: Rindopepimut

Increased EGFR amplification and/or expression [250] as well as structural mutations within the gene [251] correlate with poor clinical outcome and chemoresistance in GBM patients. As such, EGFR may be used as a potential biomarker of prognosis, as evidenced by a recent meta-analysis [252]. Numerous TKIs targeting EGFR have been examined in relation to GBM. First-generation reversible small-molecule inhibitors, Erlotinib and Gefitinib, which have been shown to be effective in treating non-small cell lung cancer [253], have also been extensively researched as therapeutic options for GBM [254]. In fact, early preclinical studies of Erlotinib and Gefitinib presented promising results when tested against GBM cell lines [255,256], but subsequent clinical studies of these ATP-competitive small molecules as monotherapeutic agents [257] or in combination therapy [258,259] were disappointing. Of note, Erlotinib is highly effective in inhibiting EGFR with kinase domain mutations that are prevalent in lung cancer [260] as opposed to its low efficacy in blocking EGFR with extracellular domain mutations that are characteristic of GBM [261,262]. 

The second-generation irreversible blocker, Afatinib, showed limited activity when used as monotherapy in unselected recurrent GBM patients [263]. However, combination therapy consisting of Afatinib plus TMZ showed a significant reduction in the proliferation and invasion potencies in U87 and U251 cells as well as GSCs isolated from U87 and U87-EGFR*vIII* cells in addition to reduced tumor burden in preclinical mouse models [264]. Despite numerous setbacks, efforts to attack GBM via EGFR targeting persist with the third-generation irreversible inhibitor AZD9291 (Osimertinib) that presents excellent blood–brain barrier penetration and promising efficacy in preclinical tests [265].

Interestingly, for the EGFR*vIII* variant, the prospect, efficacy, and safety of a peptide vaccine, Rindopepimut (CDX-110), in GBM have been tested. Rindopepimut is a 14-mer which spans the EGFR*vIII* mutation site and exerts tumor-specific activity [266]. While it showed considerable efficacy in Phase II clinical trials by demonstrating enhancement of OS, the study was terminated in Phase III due to failure to meet its primary OS endpoint [266]. However, further longitudinal analyses will help to reveal its clinical role in the treatment of GBM. 

### 6.2. IGF1R: BMS-754807, KW-2450, and Picropodophyllin

RTK inhibitors have been used as an approach to target IGF1R signaling. Initially developed for anti-IGF1R therapy, inhibitors such as BMS-754807 [267] and KW-2450 [268] showed promising activity but they often cross-react with INSR and are thus described as dual IGF1R/INSR inhibitors [269]. Another IGF1R inhibitor, picropodophyllin, showed strong efficacy by potently inhibiting IGF1R phosphorylation and signaling in addition to promoting downregulation and degradation of the receptor [270]. 

### 6.3. Abl/PDGFR/c-KIT: Imatinib (Gleevec/Glivec)

Imatinib (also known as Gleevec in USA or Glivec in Europe/Australia) is a small-molecule TKI developed to target Abl that was first used for the treatment of chronic myeloid leukemia [271]. This 2-phenyl amino pyrimidine derivative prevents the activation and function of other related tyrosine kinases (e.g., BCR-ABL, c-KIT, and PDGFR) by binding close to their ATP-binding site [271]. Several trials have searched for possible therapeutic effect of Imatinib alone against GBM but repeatedly failed to achieve significant results in reducing tumor growth or prolonging survival [272,273]. Further, one study showed that combination therapy of Imatinib and Nilotinib—a second-generation TKI that also targets PDGFR and c-KIT—did not damage GBM cells as anticipated but rather enhanced their migration and invasion via increased tyrosine phosphorylation of p130Cas, FAK, and the downstream adaptor protein paxillin [274]. Despite these disappointments, other work suggests that Imatinib can increase the radiosensitivity of human glioblastoma cells in vitro and can delay tumor growth in vivo, providing support for combination treatment with fractionated radiotherapy [275]. In sum, although effective under certain circumstances, these results clearly illustrate the limited therapeutic window of Imatinib against GBM tumors. 

A Phase II trial examining the effects of combined inhibition of VEGFRs, PDGFRs, and c-KIT looked at simultaneous administration of Bevacizumab (detailed below) and Tandutinib in patients with recurrent GBM [276]. Tandutinib is an orally administered drug that safely crosses the blood–brain barrier [277]. However, the combined therapy failed to show an improved response compared to Bevacizumab alone and was associated with neuromuscular junction toxicity [276]. 

### 6.4. VEGFs: Bevacizumab; VEGFRs: Axitinib, Cedarinib, Suritinib, and Sorafenib

Because GBMs are some of the most vascularized tumors, the VEGF receptors and their ligands play a central role as potential therapeutic targets. In fact, therapeutic intervention through VEGF inhibition by Bevacizumab (Avastin^®^) is well known. This FDA-approved recombinant humanized monoclonal antibody containing human immunoglobulin and murine complementarity-determining regions inhibits VEGF–VEGFR interactions and subsequent VEGF-mediated signaling [278]. Numerous studies have now been conducted to investigate the efficacies of small-molecular TKIs which target the VEGFR family. One such inhibitor is Axitinib (AG-013736), a selective inhibitor of VEGFR1-3 that functions by inhibiting the autophosphorylation of VEGFRs at picomolar concentrations [279], though it does inhibit PDGFRs and c-KIT as well. In a randomized Phase II study, the activity of Axitinib was compared to Bevacizumab or Lomustin, and Axitinib was determined to have good single-agent efficacy with manageable toxicity levels in patients with recurrent GBM [280]. Another pan*-VEGFR* inhibitor, Cediranib, (AZD2171) is a potent, orally administered small-molecule inhibitor that normalizes tumor blood vessels, leading to an improvement in cerebral edema [164]. Sorafenib [281] and Sunitinib (SU-11248) [282] are multi-kinase inhibitors which inhibit VEGFRs, PDGFRs, KIT, FLT3, and RET. However, Phase I studies combining Sorafenib with TMZ and radiotherapy did not substantially improve progression-free survival in GBM patients [283] and Sunitinib showed limited single-agent activity in patients with recurrent GBM [284].

### 6.5. FGFRs: AZD4547, Dovitinib, JNJ-42756493, Ponatinib, and Infigratinib

FGFRs are amongst the most altered RTKs in GBM, with many of them altered in more than one subtype in the TCGA-GBM data that we analyzed. Among FGFR1-3 inhibitors are Infigratinib (BGJ398), AZD4547, and Dovitinib, which have been investigated for their efficacies in other cancer types. AZD4547 is currently undergoing Phase I/II clinical trials (NCT02824133) in GBM patients expressing the FGFR–TACC fusion gene. BLU9931 is an FGFR4-specific inhibitor. JNJ-42756493 and Ponatinib are pan-FGFR inhibitors but show severe adverse effects [285]. A multicenter Phase II study investigating Infigratinib in FGFR-altered recurrent GBM patients revealed that Infigratinib was able to elicit a partial response or stable disease in a third of the cohort, with reversible and manageable adverse effects [286]. 

### 6.6. TrkB: ANA-12; Pan-Trk: Entrectinib and Larotrectinib

Beyond the importance of TrkB in normal neurobiology, recent studies have reported connections between aberrant BDNF/TrkB activity and various aspects of GBM. For instance, increased TrkB expression was detected in low-grade astrocytoma and GBM [287,288], while BDNF-induced activation of TrkB was found to increase the viability of tumor-initiating cells isolated from GBM [146]. Intriguingly, the ability of GBMs to make less invasive cancer cells around them more aggressive appears to be linked to the transfer of TrkB-containing exosomes, revealing a mechanism by which GBM tumors can influence their environment to promote disease progression and aggressiveness [289]. Inhibition of TrkB-associated signaling with ANA-12, which is a selective, small-molecule non-competitive TrkB antagonist that can cross the blood–brain barrier [290], has been found to limit the formation of astrocytomas [291] and the survival of GBM cancer cells [147]. These results suggest that precise TrkB inhibition might prove to be an effective strategy, possibly with fewer off-target toxicities compared with multitarget drugs in patients with GBM tumors harboring oncogenic TrkB. Discovery of more potent agents interfering with TrkB function will be necessary to fully evaluate this scenario. 

Chromosomal rearrangements causing pathological activation of *NTRK* family members (*NTRK2, NTRK3*) can confer oncogenic properties to cells by promoting proliferation, survival, and tumorigenesis (reviewed in [292]). *NTRK* oncogenic fusions with different partners have been detected with variable frequencies in many types of pediatric and adult cancers, including GBM [293], and targeting of these constitutively active chimeric receptors has been shown to provide therapeutic benefits. To date, two first-generation ATP-competitive TKIs have received FDA approval to counteract solid tumors with *NTRK* fusions: Entrectinib (also known as RXDX-101), which acts as a pan-TRK, ROS1, and ALK TKI [294], and Larotrectinib (also known as LOXO-101), which is a highly selective and potent pan-Trk inhibitor [295]. Trials for both small molecules have demonstrated efficacy against primary and secondary CNS tumors [296,297,298]. The encouraging results obtained with these two TKIs support the ongoing development of second-generation *NTRK* inhibitors (e.g., LOXO-195-BAY2731954, Repotrectinib-TPX-0005) with lower tendencies to elicit tumor resistance [299,300].

### 6.7. Tie2: Rebastinib, Bay-826, and Altiratinib

Because Ang-2 is overexpressed in GBM and also functions as a chemoattractant for TEMs that drive tumor vessel formation and metastasis, targeting the Ang-2/Tie2 signaling pathway has been shown to inhibit tumor growth and invasion in GBM [301]. Notably, the use of soluble Tie2 [302] inhibits the Ang-2/Tie2 pathway, prevents TEM recruitment to the tumor, and abrogates the heightened invasive phenotype induced by anti-VEGF therapy [175]. Selective Tie2 inhibitors such as Rebastinib have also been demonstrated to inhibit the growth, invasion, and metastasis of breast cancer cells by an “allosteric switch control” mechanism [303]. In relation to GBM, Rebastinib can successfully inhibit tumor growth and prolong survival by targeting an N-terminal truncated form of p75 (ΔNp73), which transcriptionally regulates both Tie2 and Ang-2 in concert with ETS2 proteins [304]. Another highly potent small-molecule inhibitor, Bay-826, inhibits Tie2 phosphorylation both in vitro and in murine glioma models [305]. Lastly, Altiratinib, a balanced MET/Tie2/VEGFR2 inhibitor, has been shown to substantially reduce tumor size, microvessel density, TEM infiltration, and the invasion potential of GBM cells in combination with Bevacizumab [306].

### 6.8. Other Inhibitors 

A more extensive description of TKIs as well as inhibitors that target downstream signaling components is well-covered elsewhere [307]. It is worthwhile to note that the list of inhibitors mentioned above is by no means exhaustive, and as technological progress takes place, more chemotherapeutic agents may be discovered to treat GBM.

## 7. Emerging Areas of GBM Investigation

Despite decades of research, the prognosis for GBM remains dismal, and new strategies are needed to improve disease management and treatment. Studies have revealed that the different GBM molecular subtypes show distinct responses to treatment protocols, and the integration of expression-based profiling with clinical data may further inform high-impact precision therapeutics. Indeed, in the TCGA study, patients representing the CL subtype showed the strongest response towards aggressive therapy (combination of concomitant and adjuvant TMZ with radiotherapy) compared to patients representing the ME subtype, which showed a reduced (though positive) response. On the other hand, patients with the PN subtype failed to show a significant survival advantage in response to the same treatment protocol [18]. In many ways, this can be attributed to the activation of different pathways, such as EGFR-induced Ras/MAPK. Because downstream effectors of RTKs are also prone to alterations in GBM, simultaneous targeting of multiple components of such signaling pathways will be essential to avoid drug resistance.

In recent years, the focus of GBM research has rapidly expanded to consider, with more attention, the influence of the brain environment on tumor growth [308]. An important finding which can be placed at the intersection of neuroscience and cancer biology is how the synaptic protein neuroligin-3, released in an activity-dependent manner from neurons, can act as a mitogen to stimulate the growth of malignant cells [309]. In the footsteps of this result, other studies have characterized functional glutamatergic synaptic structures between presynaptic neurons and postsynaptic tumor cells [310,311]. The most remarkable aspect from a group of separate studies that were published at the same time is the comprehensive electrophysiological, imaging, and transcriptomic evidence confirming the broad expression of synaptic effectors, including TrkB (*NTRK2),* in a variety of glioma cells, and how those components can respond to the activity of surrounding neurons to promote the progression of both primary and secondary gliomas [310,311,312]. Whether neuro-gliomal signaling could be exploited in one way or another as an attack point against GBM is an exciting possibility that will be examined closely for years to come. 

A promising new development in GBM research is the use of human organoid technology adapted to cancer modeling [313]. Notably, several publications have made clear the potential of using brain-like cerebral organoids prepared from human pluripotent stem cells to study GBM tumor formation and invasion in real time [314,315]. Indeed, our groups have recently adopted this system to demonstrate how Tie2 signaling via ShcD promotes the infiltration of GBM tumor cells [221]. Such cerebral organoid and GBM co-culture assays could be easily adapted to search for novel pharmacological agents targeting RTK signaling, such as those effective at limiting the migration of cells from GBM tumorspheres into organoids, or those able to reverse cases where the cancer cells have been allowed to penetrate deep into the organoid model prior to beginning treatment.

## 8. Conclusions and Future Perspectives

GBM remains a disease with poor prognosis and limited therapies. Given the challenges in finding effective treatments for GBM, and the propensity for tumor cells to develop resistance, future research should involve the development of inhibitors targeting non-kinase intervention points of oncogenic signaling pathways. As well, new preclinical organoid-based models should be leveraged in the next decade for enhanced testing of potential therapeutics targeting brain cancer.

## Abbreviations

BDNF CDKN2A/B	Brain-derived neurotrophic factor Cyclin-dependent kinase inhibitors 2A/B
CNA	Copy number alterations
CNS	Central nervous system
DDR ECM	Discoidin domain receptor Extracellular matrix
EGFR EMT	Epidermal growth factor receptor Epithelial-mesenchymal transition
EPH	Erythropoeitin-producing human hepatocellular receptor
FAK	Focal adhesion kinase
FLT3	FMS-like tyrosine kinase 3
GBM	Glioblastoma multiforme
G-CIMP	Glioma-CpG-island methylator phenotype
GPCR	G-protein-coupled receptor
GSC	Glioma stem cell
IDH1/2	Isocitrate dehydrogenase 1/2
IGF1R	Insulin-like growth factor 1 receptor
INSR	Insulin receptor
INSRR	Insulin-receptor-related receptor
KIT/SCFR MAPK	c-KIT/stem cell growth factor receptor Mitogen-activated protein kinase
	
MET MGMT	MNNG-HOS transforming gene O^6^-methylguanine methyltransferase
mTOR	Mammalian target of rapamycin
PDGFR	Platelet-derived growth factor receptor
PI3K PLC	Phosphatidylinositol-3-kinase Phospholipase C
PTEN	Phosphatase and tensin homolog
RET ROS1	Rearranged during transfection c-ROS oncogene 1
RPPA	Reverse Phase Protein Array
RTK	Receptor tyrosine kinase
Shc SOCE SOX2	Src homology and collagen Store operated Ca^2+^ entry Sex-determining region y-box 2
TCGA	The Cancer Genome Atlas
TEM	Tie2-expressing monocytes/macrophages
TFP TKI TMZ TRK	Trifluoperazine Tyrosine kinase inhibitor Temozolomide Tropomyosin receptor kinase
VEGFR	Vascular endothelial growth factor receptor
WHO	World Health Organization

## References

[1] Bray F., Ferlay J., Soerjomataram I., Siegel R.L., Torre L.A., Jemal A. (2018). Global cancer statistics 2018: GLOBOCAN estimates of incidence and mortality worldwide for 36 cancers in 185 countries. CA Cancer J. Clin..

[2] Goodenberger M.L., Jenkins R.B. (2012). Genetics of adult glioma. Cancer Genet..

[3] Furnari F.B., Fenton T., Bachoo R.M., Mukasa A., Stommel J.M., Stegh A., Hahn W.C., Ligon K.L., Louis D.N., Brennan C. (2007). Malignant astrocytic glioma: Genetics, biology, and paths to treatment. Genes Dev..

[4] Ohgaki H., Kleihues P. (2005). Epidemiology and etiology of gliomas. Acta Neuropathol..

[5] Louis D.N., Ohgaki H., Wiestler O.D., Cavenee W.K., Burger P.C., Jouvet A., Scheithauer B.W., Kleihues P. (2007). The 2007 WHO classification of tumours of the central nervous system. Acta Neuropathol..

[6] Ostrom Q.T., Cioffi G., Gittleman H., Patil N., Waite K., Kruchko C., Barnholtz-Sloan J.S. (2019). CBTRUS Statistical Report: Primary Brain and Other Central Nervous System Tumors Diagnosed in the United States in 2012–2016. Neuro. Oncol..

[7] Gittleman H., Ostrom Q.T., Stetson L.C., Waite K., Hodges T.R., Wright C.H., Wright J., Rubin J.B., Berens M.E., Lathia J. (2019). Sex is an important prognostic factor for glioblastoma but not for nonglioblastoma. Neuro-Oncol. Pract..

[8] Parsons D.W., Jones S., Zhang X., Lin J.C.H., Leary R.J., Angenendt P., Mankoo P., Carter H., Siu I.M., Gallia G.L. (2008). An integrated genomic analysis of human glioblastoma multiforme. Science.

[9] Jiang Y., Uhrbom L. (2012). On the origin of glioma. Upsala J. Med. Sci..

[10] Ohgaki H., Kleihues P. (2013). The definition of primary and secondary glioblastoma. Clin. Cancer Res..

[11] Louis D.N., Perry A., Reifenberger G., von Deimling A., Figarella-Branger D., Cavenee W.K., Ohgaki H., Wiestler O.D., Kleihues P., Ellison D.W. (2016). The 2016 World Health Organization Classification of Tumors of the Central Nervous System: A summary. Acta Neuropathol..

[12] Newlands E.S., Stevens M.F.G., Wedge S.R., Wheelhouse R.T., Brock C. (1997). Temozolomide: A review of its discovery, chemical properties, pre-clinical development and clinical trials. Cancer Treat. Rev..

[13] Stupp R., Mason W.P., Van Den Bent M.J., Weller M., Fisher B., Taphoorn M.J.B., Belanger K., Brandes A.A., Marosi C., Bogdahn U. (2005). Radiotherapy plus concomitant and adjuvant temozolomide for glioblastoma. N. Engl. J. Med..

[14] Stupp R., Hegi M.E., Mason W.P., van den Bent M.J., Taphoorn M.J., Janzer R.C., Ludwin S.K., Allgeier A., Fisher B., Belanger K. (2009). Effects of radiotherapy with concomitant and adjuvant temozolomide versus radiotherapy alone on survival in glioblastoma in a randomised phase III study: 5-year analysis of the EORTC-NCIC trial. Lancet Oncol..

[15] Hart M.G., Garside R., Rogers G., Stein K., Grant R. (2013). Temozolomide for high grade glioma. Cochrane Database Syst. Rev..

[16] Freije W.A., Castro-Vargas F.E., Fang Z., Horvath S., Cloughesy T., Liau L.M., Mischel P.S., Nelson S.F. (2004). Gene expression profiling of gliomas strongly predicts survival. Cancer Res..

[17] Phillips H.S., Kharbanda S., Chen R., Forrest W.F., Soriano R.H., Wu T.D., Misra A., Nigro J.M., Colman H., Soroceanu L. (2006). Molecular subclasses of high-grade glioma predict prognosis, delineate a pattern of disease progression, and resemble stages in neurogenesis. Cancer Cell.

[18] Verhaak R.G.W., Hoadley K.A., Purdom E., Wang V., Qi Y., Wilkerson M.D., Miller C.R., Ding L., Golub T., Mesirov J.P. (2010). Integrated Genomic Analysis Identifies Clinically Relevant Subtypes of Glioblastoma Characterized by Abnormalities in PDGFRA, IDH1, EGFR, and NF1. Cancer Cell.

[19] Wang Q., Hu B., Hu X., Kim H., Squatrito M., Scarpace L., de Carvalho A.C., Lyu S., Li P., Li Y. (2017). Tumor Evolution of Glioma-Intrinsic Gene Expression Subtypes Associates with Immunological Changes in the Microenvironment. Cancer Cell.

[20] Brennan C.W., Verhaak R.G.W., McKenna A., Campos B., Noushmehr H., Salama S.R., Zheng S., Chakravarty D., Sanborn J.Z., Berman S.H. (2013). The somatic genomic landscape of glioblastoma. Cell.

[21] Gao J., Aksoy B.A., Dogrusoz U., Dresdner G., Gross B., Sumer S.O., Sun Y., Jacobsen A., Sinha R., Larsson E. (2013). Integrative analysis of complex cancer genomics and clinical profiles using the cBioPortal. Sci. Signal..

[22] Gómez-Rubio V. (2017). ggplot2—Elegant Graphics for Data Analysis (2nd Edition). J. Stat. Softw..

[23] McLendon R., Friedman A., Bigner D., Van Meir E.G., Brat D.J., Mastrogianakis G.M., Olson J.J., Mikkelsen T., Lehman N., Aldape K. (2008). Comprehensive genomic characterization defines human glioblastoma genes and core pathways. Nature.

[24] Noushmehr H., Weisenberger D.J., Diefes K., Phillips H.S., Pujara K., Berman B.P., Pan F., Pelloski C.E., Sulman E.P., Bhat K.P. (2010). Identification of a CpG Island Methylator Phenotype that Defines a Distinct Subgroup of Glioma. Cancer Cell.

[25] Malta T.M., De Souza C.F., Sabedot T.S., Silva T.C., Mosella M.S., Kalkanis S.N., Snyder J., Castro A.V.B., Noushmehr H. (2018). Glioma CpG island methylator phenotype (G-CIMP): Biological and clinical implications. Neuro. Oncol..

[26] Martinez R., Schackert G., Yaya-Tur R., Rojas-Marcos I., Herman J.G., Esteller M. (2007). Frequent hypermethylation of the DNA repair gene MGMT in long-term survivors of glioblastoma multiforme. J. Neurooncol..

[27] Erasimus H., Gobin M., Niclou S., Van Dyck E. (2016). DNA repair mechanisms and their clinical impact in glioblastoma. Mutat. Res. Mutat. Res..

[28] Kitange G.J., Carlson B.L., Schroeder M.A., Grogan P.T., Lamont J.D., Decker P.A., Wu W., James C.D., Sarkaria J.N. (2009). Induction of MGMT expression is associated with temozolomide resistance in glioblastoma xenografts. Neuro. Oncol..

[29] Ichimura K., Pearson D.M., Kocialkowski S., Bäcklund L.M., Chan R., Jones D.T.W., Collins V.P. (2009). IDH1 mutations are present in the majority of common adult gliomas but rare in primary glioblastomas. Neuro. Oncol..

[30] Xu X., Zhao J., Xu Z., Peng B., Huang Q., Arnold E., Ding J. (2004). Structures of human cytosolic NADP-dependent isocitrate dehydrogenase reveal a novel self-regulatory mechanism of activity. J. Biol. Chem..

[31] Watanabe T., Nobusawa S., Kleihues P., Ohgaki H. (2009). IDH1 mutations are early events in the development of astrocytomas and oligodendrogliomas. Am. J. Pathol..

[32] Yan H., Parsons D.W., Jin G., McLendon R., Rasheed B.A., Yuan W., Kos I., Batinic-Haberle I., Jones S., Riggins G.J. (2009). IDH1 and IDH2 mutations in gliomas. N. Engl. J. Med..

[33] Zhao S., Lin Y., Xu W., Jiang W., Zhai Z., Wang P., Yu W., Li Z., Gong L., Peng Y. (2009). Glioma-derived mutations in IDH1 dominantly inhibit IDH1 catalytic activity and induce HIF-1α. Science.

[34] Butler M., Pongor L., Su Y.T., Xi L., Raffeld M., Quezado M., Trepel J., Aldape K., Pommier Y., Wu J. (2020). MGMT Status as a Clinical Biomarker in Glioblastoma. Trends Cancer.

[35] Gullotta F., Schindler F., Schmutzler R., Weeks-Seifert A. (1985). GFAP in Brain Tumor Diagnosis: Possibilities and Limitations. Pathol. Res. Pract..

[36] Kajiwara K., Orita T., Nishizaki T., Kamiryo T., Nakayama H., Ito H. (1992). Glial fibrillary acidic protein (GFAP) expression and nucleolar organizer regions (NORs) in human gliomas. Brain Res..

[37] Ligon K.L., Alberta J.A., Kho A.T., Weiss J., Kwaan M.R., Nutt C.L., Louis D.N., Stiles C.D., Rowitch D.H. (2004). The oligodendroglial lineage marker OLIG2 is universally expressed in diffuse gliomas. J. Neuropathol. Exp. Neurol..

[38] Mokhtari K., Paris S., Aguirre-Cruz L., Privat N., Crinière E., Marie Y., Hauw J.J., Kujas M., Rowitch D., Hoang-Xuan K. (2005). Olig2 expression, GFAP, p53 and 1p loss analysis contribute to glioma subclassification. Neuropathol. Appl. Neurobiol..

[39] Ma Y.H., Mentlein R., Knerlich F., Kruse M.L., Mehdorn H.M., Held-Feindt J. (2008). Expression of stem cell markers in human astrocytomas of different WHO grades. J. Neurooncol..

[40] Takenobu H., Shimozato O., Nakamura T., Ochiai H., Yamaguchi Y., Ohira M., Nakagawara A., Kamijo T. (2011). CD133 suppresses neuroblastoma cell differentiation via signal pathway modification. Oncogene.

[41] Berezovsky A.D., Poisson L.M., Cherba D., Webb C.P., Transou A.D., Lemke N.W., Hong X., Hasselbach L.A., Irtenkauf S.M., Mikkelsen T. (2014). Sox2 promotes malignancy in glioblastoma by regulating plasticity and astrocytic differentiation. Neoplasia.

[42] Hattermann K., Flüh C., Engel D., Mehdorn H.M., Synowitz M., Mentlein R., Held-Feindt J. (2016). Stem cell markers in glioma progression and recurrence. Int. J. Oncol..

[43] Rashidian J., Copaciu R., Su Q., Merritt B., Johnson C., Yahyabeik A., French E., Cummings K. (2017). Generation and Performance of R132H Mutant IDH1 Rabbit Monoclonal Antibody. Antibodies.

[44] Agarwal S., Sharma M.C., Jha P., Pathak P., Suri V., Sarkar C., Chosdol K., Suri A., Kale S.S., Mahapatra A.K. (2013). Comparative study of IDH1 mutations in gliomas by immunohistochemistry and DNA sequencing. Neuro. Oncol..

[45] Reuss D.E., Sahm F., Schrimpf D., Wiestler B., Capper D., Koelsche C., Schweizer L., Korshunov A., Jones D.T.W., Hovestadt V. (2015). ATRX and IDH1-R132H immunohistochemistry with subsequent copy number analysis and IDH sequencing as a basis for an “integrated” diagnostic approach for adult astrocytoma, oligodendroglioma and glioblastoma. Acta Neuropathol..

[46] Hanahan D., Weinberg R.A. (2011). Hallmarks of cancer: The next generation. Cell.

[47] Giese A., Loo M.A., Tran N., Haskett D., Coons S.W., Berens M.E. (1996). Dichotomy of astrocytoma migration and proliferation. Int. J. Cancer.

[48] Rajapakse V.N., Herrada S., Lavi O. (2020). Phenotype stability under dynamic brain-tumor environment stimuli maps glioblastoma progression in patients. Sci. Adv..

[49] Monteiro A., Hill R., Pilkington G., Madureira P. (2017). The Role of Hypoxia in Glioblastoma Invasion. Cells.

[50] Velásquez C., Mansouri S., Mora C., Nassiri F., Suppiah S., Martino J., Zadeh G., Fernández-Luna J.L. (2019). Molecular and Clinical Insights into the Invasive Capacity of Glioblastoma Cells. J. Oncol..

[51] Bindal A.K., Hammoud M., Shi W.M., Wu S.Z., Sawaya R., Rao J.S. (1994). Prognostic significance of proteolytic enzymes in human brain tumors. J. Neurooncol..

[52] Iwadate Y. (2016). Epithelial-mesenchymal transition in glioblastoma progression. Oncol. Lett..

[53] Brabletz T., Jung A., Spaderna S., Hlubek F., Kirchner T. (2005). Migrating cancer stem cells—An integrated concept of malignant tumour progression. Nat. Rev. Cancer.

[54] Ho I.A.W., Shim W.S.N. (2017). Contribution of the microenvironmental niche to glioblastoma heterogeneity. BioMed Res. Int..

[55] Prager B.C., Xie Q., Bao S., Rich J.N. (2019). Cancer Stem Cells: The Architects of the Tumor Ecosystem. Cell Stem Cell.

[56] Gilbertson R.J., Rich J.N. (2007). Making a tumour’s bed: Glioblastoma stem cells and the vascular niche. Nat. Rev. Cancer.

[57] Aderetti D.A., Hira V.V.V., Molenaar R.J., van Noorden C.J.F. (2018). The hypoxic peri-arteriolar glioma stem cell niche, an integrated concept of five types of niches in human glioblastoma. Biochim. Biophys. Acta Rev. Cancer.

[58] Bouwens van der Vlis T.A.M., Kros J.M., Mustafa D.A.M., van Wijck R.T.A., Ackermans L., van Hagen P.M., van der Spek P.J. (2018). The complement system in glioblastoma multiforme. Acta Neuropathol. Commun..

[59] Dirkse A., Golebiewska A., Buder T., Nazarov P.V., Muller A., Poovathingal S., Brons N.H.C., Leite S., Sauvageot N., Sarkisjan D. (2019). Stem cell-associated heterogeneity in Glioblastoma results from intrinsic tumor plasticity shaped by the microenvironment. Nat. Commun..

[60] Schiffer D., Mellai M., Bovio E., Bisogno I., Casalone C., Annovazzi L. (2018). Glioblastoma niches: From the concept to the phenotypical reality. Neurol. Sci..

[61] Vescovi A.L., Galli R., Reynolds B.A. (2006). Brain tumour stem cells. Nat. Rev. Cancer.

[62] Lathia J.D., Mack S.C., Mulkearns-Hubert E.E., Valentim C.L.L., Rich J.N. (2015). Cancer stem cells in glioblastoma. Genes Dev..

[63] Cheng L., Wu Q., Guryanova O.A., Huang Z., Huang Q., Rich J.N., Bao S. (2011). Elevated invasive potential of glioblastoma stem cells. Biochem. Biophys. Res. Commun..

[64] Bao S., Wu Q., McLendon R.E., Hao Y., Shi Q., Hjelmeland A.B., Dewhirst M.W., Bigner D.D., Rich J.N. (2006). Glioma stem cells promote radioresistance by preferential activation of the DNA damage response. Nature.

[65] Suvà M.L., Tirosh I. (2020). The Glioma Stem Cell Model in the Era of Single-Cell Genomics. Cancer Cell.

[66] Patel A.P., Tirosh I., Trombetta J.J., Shalek A.K., Gillespie S.M., Wakimoto H., Cahill D.P., Nahed B.V., Curry W.T., Martuza R.L. (2014). Single-cell RNA-seq highlights intratumoral heterogeneity in primary glioblastoma. Science.

[67] Neftel C., Laffy J., Filbin M.G., Hara T., Shore M.E., Rahme G.J., Richman A.R., Silverbush D., Shaw M.L., Hebert C.M. (2019). An Integrative Model of Cellular States, Plasticity, and Genetics for Glioblastoma. Cell.

[68] Hardee M.E., Zagzag D. (2012). Mechanisms of glioma-associated neovascularization. Am. J. Pathol..

[69] Zadeh G., Koushan K., Pillo L., Shannon P., Guha A. (2004). Role of Ang1 and its interaction with VEGF-A in astrocytomas. J. Neuropathol. Exp. Neurol..

[70] Zagzag D., Hooper A., Friedlander D.R., Chan W., Holash J., Wiegand S.J., Yancopoulos G.D., Grumet M. (1999). In situ expression of angiopoietins in astrocytomas identifies angiopoietin-2 as an early marker of tumor angiogenesis. Exp. Neurol..

[71] Ricci-Vitiani L., Pallini R., Biffoni M., Todaro M., Invernici G., Cenci T., Maira G., Parati E.A., Stassi G., Larocca L.M. (2010). Tumour vascularization via endothelial differentiation of glioblastoma stem-like cells. Nature.

[72] Treps L., Perret R., Edmond S., Ricard D., Gavard J. (2017). Glioblastoma stem-like cells secrete the pro-angiogenic VEGF-A factor in extracellular vesicles. J. Extracell. Vesicles.

[73] Salajegheh A. (2016). Angiogenesis in Health, Disease and Malignancy.

[74] Onishi M., Ichikawa T., Kurozumi K., Date I. (2011). Angiogenesis and invasion in glioma. Brain Tumor Pathol..

[75] Brahimi-Horn C., Berra E., Pouysségur J. (2001). Hypoxia: The tumor’s gateway to progression along the angiogenic pathway. Trends Cell Biol..

[76] Ferrara N. (2004). Vascular Endothelial Growth Factor: Basic Science and Clinical Progress. Endocr. Rev..

[77] Chambless L.B., Parker S.L., Hassam-Malani L., McGirt M.J., Thompson R.C. (2012). Type 2 diabetes mellitus and obesity are independent risk factors for poor outcome in patients with high-grade glioma. J. Neurooncol..

[78] Gong Y., Ma Y., Sinyuk M., Loganathan S., Thompson R.C., Sarkaria J.N., Chen W., Lathia J.D., Mobley B.C., Clark S.W. (2016). Insulin-mediated signaling promotes proliferation and survival of glioblastoma through Akt activation. Neuro. Oncol..

[79] Zhang C., Moore L.M., Li X., Yung W.K.A., Zhang W. (2013). IDH1/2 mutations target a key hallmark of cancer by deregulating cellular metabolism in glioma. Neuro. Oncol..

[80] Agnihotri S., Zadeh G. (2016). Metabolic reprogramming in glioblastoma: The influence of cancer metabolism on epigenetics and unanswered questions. Neuro. Oncol..

[81] Snuderl M., Fazlollahi L., Le L.P., Nitta M., Zhelyazkova B.H., Davidson C.J., Akhavanfard S., Cahill D.P., Aldape K.D., Betensky R.A. (2011). Mosaic amplification of multiple receptor tyrosine kinase genes in glioblastoma. Cancer Cell.

[82] Van der Geer P., Hunter T., Lindberg R.A. (1994). Receptor Protein-Tyrosine Kinases and Their Signal Transduction Pathways. Annu. Rev. Cell Biol..

[83] Schlessinger J. (2000). Cell signaling by receptor tyrosine kinases. Cell.

[84] Joshi G., Singh P.K., Negi A., Rana A., Singh S., Kumar R. (2015). Growth factors mediated cell signalling in prostate cancer progression: Implications in discovery of anti-prostate cancer agents. Chem. Biol. Interact..

[85] Normanno N., De Luca A., Bianco C., Strizzi L., Mancino M., Maiello M.R., Carotenuto A., De Feo G., Caponigro F., Salomon D.S. (2006). Epidermal growth factor receptor (EGFR) signaling in cancer. Gene.

[86] Roberts J.G., Williams M., Henk J.M., Bligh A.S., Baum M. (1975). The hypronosticon test in breast cancer. Clin.Oncol..

[87] Black P.C., Brown G.A., Dinney C.P., Kassouf W., Inamoto T., Arora A., Gallagher D., Munsell M.F., Bar-Eli M., McConkey D.J. (2011). Receptor heterodimerization: A new mechanism for platelet-derived growth factor induced resistance to anti-epidermal growth factor receptor therapy for bladder cancer. J. Urol..

[88] Erikson J., Griffin C.A., Ar-Rushdi A., Valtieri M., Hoxie J., Finan J., Emanuel B.S., Rovera G., Nowell P.C., Croce C.M. (1986). Heterogeneity of chromosome 22 breakpoint in Philadelphia-positive (Ph+) acute lymphocytic leukemia. Proc. Natl. Acad. Sci. USA.

[89] Fantl W.J., Johnson D.E., Williams L.T. (1993). Signalling by Receptor Tyrosine Kinases. Annu. Rev. Biochem..

[90] Carvalho-Silva D., Pierleoni A., Pignatelli M., Ong C.K., Fumis L., Karamanis N., Carmona M., Faulconbridge A., Hercules A., McAuley E. (2019). Open Targets Platform: New developments and updates two years on. Nucleic Acids Res..

[91] Manning G., Whyte D.B., Martinez R., Hunter T., Sudarsanam S. (2002). The protein kinase complement of the human genome. Science.

[92] Metz K.S., Deoudes E.M., Berginski M.E., Jimenez-Ruiz I., Aksoy B.A., Hammerbacher J., Gomez S.M., Phanstiel D.H. (2018). Coral: Clear and Customizable Visualization of Human Kinome Data. Cell Syst..

[93] Burgess A.W. (2008). EGFR family: Structure physiology signalling and therapeutic target. Growth Factors.

[94] Plowman G.D., Culouscou J.M., Whitney G.S., Green J.M., Carlton G.W., Foy L., Neubauer M.G., Shoyab M. (1993). Ligand-specific activation of HER4/p180erbB4, a fourth member of the epidermal growth factor receptor family. Proc. Natl. Acad. Sci. USA.

[95] Plowman G.D., Whitney G.S., Neubauer M.G., Green J.M., McDonald V.L., Todaro G.J., Shoyab M. (1990). Molecular cloning and expression of an additional epidermal growth factor receptor-related gene. Proc. Natl. Acad. Sci. USA.

[96] Stern D.F., Heffernan P.A., Weinberg R.A. (1986). P185, a Product of the Neu Proto-Oncogene, Is a Receptorlike Protein Associated with Tyrosine Kinase Activity. Mol. Cell. Biol..

[97] Bargmann C.I., Weinberg R.A. (1988). Increased tyrosine kinase activity associated with the protein encoded by the activated neu oncogene. Proc. Natl. Acad. Sci. USA.

[98] Schlessinger J. (2002). Ligand-induced, receptor-mediated dimerization and activation of EGF receptor. Cell.

[99] Blume-Jensen P., Hunter T. (2001). Oncogenic kinase signalling. Nature.

[100] Hatanpaa K.J., Burma S., Zhao D., Habib A.A. (2010). Epidermal growth factor receptor in glioma: Signal transduction, neuropathology, imaging, and radioresistance1. Neoplasia.

[101] Rowinsky E.K. (2004). The erbB Family: Targets for Therapeutic Development Against Cancer and Therapeutic Strategies Using Monoclonal Antibodies and Tyrosine Kinase Inhibitors. Annu. Rev. Med..

[102] Zadeh G., Bhat K.L., Aldape K. (2013). EGFR and EGFRvIII in Glioblastoma: Partners in Crime. Cancer Cell.

[103] Fan Q.W., Cheng C.K., Gustafson W.C., Charron E., Zipper P., Wong R.A., Chen J., Lau J., Knobbe-Thomsen C., Weller M. (2013). EGFR Phosphorylates Tumor-Derived EGFRvIII Driving STAT3/5 and Progression in Glioblastoma. Cancer Cell.

[104] Koboldt D.C., Fulton R.S., McLellan M.D., Schmidt H., Kalicki-Veizer J., McMichael J.F., Fulton L.L., Dooling D.J., Ding L., Mardis E.R. (2012). Comprehensive molecular portraits of human breast tumours. Nature.

[105] Schlegel J., Stumm G., Brändle K., Merdes A., Mechtersheimer G., Hynes N.E., Kiessling M. (1994). Amplification and differential expression of members of the erbB-gene family in human glioblastoma. J. Neurooncol..

[106] Zhang C., Burger M.C., Jennewein L., Genßler S., Schönfeld K., Zeiner P., Hattingen E., Harter P.N., Mittelbronn M., Tonn T. (2016). ErbB2/HER2-Specific NK Cells for Targeted Therapy of Glioblastoma. J. Natl. Cancer Inst..

[107] Andersson U., Guo D., Malmer B., Bergenheim A.T., Brännström T., Hedman H., Henriksson R. (2004). Epidermal growth factor receptor family (EGFR, ErbB2-4) in gliomas and meningiomas. Acta Neuropathol..

[108] Birchmeier C. (2009). ErbB receptors and the development of the nervous system. Exp. Cell Res..

[109] Donoghue J.F., Kerr L.T., Alexander N.W., Greenall S.A., Longano A.B., Gottardo N.G., Wang R., Tabar V., Adams T.E., Mischel P.S. (2018). Activation of ERBB4 in glioblastoma can contribute to increased tumorigenicity and influence therapeutic response. Cancers.

[110] Belfiore A., Malaguarnera R., Vella V., Lawrence M.C., Sciacca L., Frasca F., Morrione A., Vigneri R. (2017). Insulin receptor isoforms in physiology and disease: An updated view. Endocr. Rev..

[111] Taniguchi C.M., Emanuelli B., Kahn C.R. (2006). Critical nodes in signalling pathways: Insights into insulin action. Nat. Rev. Mol. Cell Biol..

[112] Payankaulam S., Raicu A.M., Arnosti D.N. (2019). Transcriptional regulation of INSR, the insulin receptor gene. Genes.

[113] Trojan J., Cloix J.F., Ardourel M.Y., Chatel M., Anthony D.D. (2007). Insulin-like growth factor type I biology and targeting in malignant gliomas. Neuroscience.

[114] Deyev I.E., Rzhevsky D.I., Berchatova A.A., Serova O.V., Popova N.V., Murashev A.N., Petrenko A.G. (2011). Deficient Response to Experimentally Induced Alkalosis in Mice with the Inactivated insrr Gene. Acta Nat..

[115] Maris C., D’Haene N., Trépant A.L., Le Mercier M., Sauvage S., Allard J., Rorive S., Demetter P., Decaestecker C., Salmon I. (2015). IGF-IR: A new prognostic biomarker for human glioblastoma. Br. J. Cancer.

[116] Girnita L., Worrall C., Takahashi S.I., Seregard S., Girnita A. (2014). Something old, something new and something borrowed: Emerging paradigm of insulin-like growth factor type 1 receptor (IGF-1R) signaling regulation. Cell. Mol. Life Sci..

[117] Fruttiger M., Karlsson L., Hall A.C., Abramsson A., Calver A.R., Boström H., Willetts K., Bertold C.H., Heath J.K., Betsholtz C. (1999). Defective oligodendrocyte development and severe hypomyelination in PDGF-A knockout mice. Development.

[118] Hellström M., Kalén M., Lindahl P., Abramsson A., Betsholtz C. (1999). Role of PDGF-B and PDGFR-β in recruitment of vascular smooth muscle cells and pericytes during embryonic blood vessel formation in the mouse. Development.

[119] Ozawa T., Brennan C.W., Wang L., Squatrito M., Sasayama T., Nakada M., Huse J.T., Pedraza A., Utsuki S., Yasui Y. (2010). PDGFRA gene rearrangements are frequent genetic events in PDGFRA-amplified glioblastomas. Genes Dev..

[120] Kim Y., Kim E., Wu Q., Guryanova O., Hitomi M., Lathia J.D., Serwanski D., Sloan A.E., Weil R.J., Lee J. (2012). Platelet-derived growth factor receptors differentially inform intertumoral and intratumoral heterogeneity. Genes Dev..

[121] Matsui J., Wakabayashi T., Asada M., Yoshimatsu K., Okada M. (2004). Stem Cell Factor/c-kit Signaling Promotes the Survival, Migration, and Capillary Tube Formation of Human Umbilical Vein Endothelial Cells. J. Biol. Chem..

[122] Gomes A.L., Reis-Filho J.S., Lopes J.M., Martinho O., Lambros M.B.K., Martins A., Schmitt F., Pardal F., Reis R.M. (2007). Molecular alterations of KIT oncogene in gliomas. Cell. Oncol..

[123] Grafone T., Palmisano M., Nicci C., Storti S. (2012). An overview on the role of FLT3-tyrosine kinase receptor in acute myeloid leukemia: Biology and treatment. Oncol. Rev..

[124] Eßbach C., Andrae N., Pachow D., Warnke J.P., Wilisch-Neumann A., Kirches E., Mawrin C. (2013). Abundance of Flt3 and its ligand in astrocytic tumors. Onco. Targets Ther..

[125] Shibuya M., Yamaguchi S., Yamane A., Ikeda T., Tojo A., Matsushime H., Sato M. (1990). Nucleotide sequence and expression of a novel human receptor-type tyrosine kinase gene (flt) closely relatd to the fms family. Oncogene.

[126] Matthews W., Jordan C.T., Gavin M., Jenkins N.A., Copeland N.G., Lemischka I.R. (1991). A receptor tyrosine kinase cDNA isolated from a population of enriched primitive hematopoietic cells and exhibiting close genetic linkage to c-kit. Proc. Natl. Acad. Sci. USA.

[127] Galland F., Karamysheva A., Pebusque M.J., Borg J.P., Rottapel R., Dubreuil P., Rosnet O., Birnbaum D. (1993). The FLT4 gene encodes a transmembrane tyrosine kinase related to the vascular endothelial growth factor receptor. Oncogene.

[128] Shibuya M. (2011). Vascular Endothelial Growth Factor (VEGF) and Its Receptor (VEGFR) Signaling in Angiogenesis: A Crucial Target for Anti- and Pro-Angiogenic Therapies. Genes Cancer.

[129] Takahashi H., Shibuya M. (2005). The vascular endothelial growth factor (VEGF)/VEGF receptor system and its role under physiological and pathological conditions. Clin. Sci..

[130] Loureiro L.V.M., Neder L., Callegaro-Filho D., de Oliveira Koch L., Stavale J.N., Malheiros S.M.F. (2020). The immunohistochemical landscape of the VEGF family and its receptors in glioblastomas. Surg. Exp. Pathol..

[131] Guarnaccia L., Navone S.E., Trombetta E., Cordiglieri C., Cherubini A., Crisà F.M., Rampini P., Miozzo M., Fontana L., Caroli M. (2018). Angiogenesis in human brain tumors: Screening of drug response through a patient-specific cell platform for personalized therapy. Sci. Rep..

[132] Siveen K.S., Prabhu K., Krishnankutty R., Kuttikrishnan S., Tsakou M., Alali F.Q., Dermime S., Mohammad R.M., Uddin S. (2017). Vascular Endothelial Growth Factor (VEGF) Signaling in Tumour Vascularization: Potential and Challenges. Curr. Vasc. Pharmacol..

[133] Zimna A., Kurpisz M. (2015). Hypoxia-Inducible factor-1 in physiological and pathophysiological angiogenesis: Applications and therapies. BioMed Res. Int..

[134] Turner N., Grose R. (2010). Fibroblast growth factor signalling: From development to cancer. Nat. Rev. Cancer.

[135] Collette J.C., Choubey L., Smith K.M. (2017). Glial and stem cell expression of murine fibroblast growth factor receptor 1 in the embryonic and perinatal nervous system. PeerJ.

[136] Kang W., Hébert J.M. (2015). FGF signaling is necessary for neurogenesis in young mice and sufficient to reverse its decline in old mice. J. Neurosci..

[137] Touat M., Ileana E., Postel-Vinay S., André F., Soria J.C. (2015). Targeting FGFR signaling in cancer. Clin. Cancer Res..

[138] Darmanis S., Sloan S.A., Croote D., Mignardi M., Chernikova S., Samghababi P., Zhang Y., Neff N., Kowarsky M., Caneda C. (2017). Single-Cell RNA-Seq Analysis of Infiltrating Neoplastic Cells at the Migrating Front of Human Glioblastoma. Cell Rep..

[139] Huang E.J., Reichardt L.F. (2003). Trk Receptors: Roles in Neuronal Signal Transduction. Annu. Rev. Biochem..

[140] Cohen-Cory S., Kidane A.H., Shirkey N.J., Marshak S. (2010). Brain-derived neurotrophic factor and the development of structural neuronal connectivity. Dev. Neurobiol..

[141] Segal R.A. (2003). Selectivity in neurotrophin signaling: Theme and variations. Annu. Rev. Neurosci..

[142] Klein R., Jing S., Nanduri V., O’Rourke E., Barbacid M. (1991). The trk proto-oncogene encodes a receptor for nerve growth factor. Cell.

[143] Singer H.S., Hansen B., Martinie D., Karp C.L. (1999). Mitogenesis in glioblastoma multiforme cell lines: A role for NGF and its TrkA receptors. J. Neurooncol..

[144] Lu B., Figurov A. (1997). Role of neurotrophins in synapse development and plasticity. Rev. Neurosci..

[145] Guo W., Nagappan G., Lu B. (2018). Differential effects of transient and sustained activation of BDNF-TrkB signaling. Dev. Neurobiol..

[146] Lawn S., Krishna N., Pisklakova A., Qu X., Fenstermacher D.A., Fournier M., Vrionis F.D., Tran N., Chan J.A., Kenchappa R.S. (2015). Neurotrophin Signaling via TrkB and TrkC Receptors Promotes the Growth of Brain Tumor-initiating Cells. J. Biol. Chem..

[147] Pinheiro K.V., Alves C., Buendia M., Gil M.S., Thomaz A., Schwartsmann G., De Farias C.B., Roesler R. (2017). Targeting tyrosine receptor kinase B in gliomas. Neuro. Oncol..

[148] Wu G., Broniscer A., McEachron T.A., Lu C., Paugh B.S., Becksfort J., Qu C., Ding L., Huether R., Parker M. (2012). Somatic histone H3 alterations in pediatric diffuse intrinsic pontine gliomas and non-brainstem glioblastomas. Nat. Genet..

[149] Petrini I. (2015). Biology of MET: A double life between normal tissue repair and tumor progression. Ann. Transl. Med..

[150] Bladt F., Riethmacher D., Isenmann S., Aguzzi A., Birchmeier C. (1995). Essential role for the c-met receptor in the migration of myogenic precursor cells into the limb bud. Nature.

[151] Cheng F., Guo D. (2019). MET in glioma: Signaling pathways and targeted therapies. J. Exp. Clin. Cancer Res..

[152] Li Y., Li A., Glas M., Lal B., Ying M., Sang Y., Xia S., Trageser D., Guerrero-Cázares H., Eberhart C.G. (2011). c-Met signaling induces a reprogramming network and supports the glioblastoma stem-like phenotype. Proc. Natl. Acad. Sci. USA.

[153] Rajadurai C.V., Havrylov S., Zaoui K., Vaillancourt R., Stuible M., Naujokas M., Zuo D., Tremblay M.L., Park M. (2012). Met receptor tyrosine kinase signals through a cortactin-Gab1 scaffold complex, to mediate invadopodia. J. Cell Sci..

[154] Petterson S.A., Dahlrot R.H., Hermansen S.K., Munthe S.K.A., Gundesen M.T., Wohlleben H., Rasmussen T., Beier C.P., Hansen S., Kristensen B.W. (2015). High levels of c-Met is associated with poor prognosis in glioblastoma. J. Neurooncol..

[155] Kong D.S., Song S.Y., Kim D.H., Kyeung M.J., Yoo J.S., Jong S.K., Seung M.D., Suh Y.L., Lee J.I., Park K. (2009). Prognostic significance of c-Met expression in glioblastomas. Cancer.

[156] Gale N.W., Holland S.J., Valenzuela D.M., Flenniken A., Pan L., Ryan T.E., Henkemeyer M., Strebhardt K., Hirai H., Wilkinson D.G. (1996). Eph receptors and ligands comprise two major specificity subclasses and are reciprocally compartmentalized during embryogenesis. Neuron.

[157] Pasquale E.B. (2008). Eph-Ephrin Bidirectional Signaling in Physiology and Disease. Cell.

[158] Yamaguchi Y., Pasquale E.B. (2004). Eph receptors in the adult brain. Curr. Opin. Neurobiol..

[159] Wilkinson D.G. (2000). Eph receptors and ephrins: Regulators of guidance and assembly. Int. Rev. Cytol..

[160] Wilkinson D.G. (2001). Multiple roles of eph receptors and ephrins in neural development. Nat. Rev. Neurosci..

[161] Ferluga S., Tomé C.M.L., Herpai D.M., D’Agostino R., Debinski W. (2016). Simultaneous targeting of Eph receptors in glioblastoma. Oncotarget.

[162] Binda E., Visioli A., Giani F., Lamorte G., Copetti M., Pitter K.L., Huse J.T., Cajola L., Zanetti N., DiMeco F. (2012). The EphA2 Receptor Drives Self-Renewal and Tumorigenicity in Stem-like Tumor-Propagating Cells from Human Glioblastomas. Cancer Cell.

[163] Day B.W., Stringer B.W., Al-Ejeh F., Ting M.J., Wilson J., Ensbey K.S., Jamieson P.R., Bruce Z.C., Lim Y.C., Offenhäuser C. (2013). EphA3 Maintains Tumorigenicity and Is a Therapeutic Target in Glioblastoma Multiforme. Cancer Cell.

[164] Qazi M.A., Vora P., Venugopal C., Adams J., Singh M., Hu A., Gorelik M., Subapanditha M.K., Savage N., Yang J. (2018). Cotargeting ephrin receptor tyrosine kinases A2 and A3 in cancer stem cells reduces growth of recurrent glioblastoma. Cancer Res..

[165] Dumont D.J., Yamaguchi T.P., Conlon R.A., Rossant J., Breitman M.L. (1992). Tek, a novel tyrosine kinase gene located on mouse chromosome 4, is expressed in endothelial cells and their presumptive precursors. Oncogene.

[166] Jones N., Iljin K., Dumont D.J., Alitalo K. (2001). Tie receptors: New modulators of angiogenic and lymphangiogenic responses. Nat. Rev. Mol. Cell Biol..

[167] Lee O.H., Xu J., Fueyo J., Fuller G.N., Aldape K.D., Alonso M.M., Piao Y., Liu T.J., Lang F.F., Bekele B.N. (2006). Expression of the receptor tyrosine kinase Tie2 in neoplastic glial cells is associated with integrin β1-dependent adhesion to the extracellular matrix. Mol. Cancer Res..

[168] Serrano Cardona L., Muñoz Mata E. (2013). Paraninfo Digital. Early Hum. Dev..

[169] Teichert-Kuliszewska K., Maisonpierre P.C., Jones N., Campbell A.I.M., Master Z., Bendeck M.P., Alitalo K., Dumont D.J., Yancopoulos G.D., Stewart D.J. (2001). Biological action of angiopoietin-2 in a fibrin matrix model of angiogenesis is associated with activation of Tie2. Cardiovasc. Res..

[170] Daly C., Eichten A., Castanaro C., Pasnikowski E., Adler A., Lalani A.S., Papadopoulos N., Kyle A.H., Minchinton A.I., Yancopoulos G.D. (2013). Angiopoietin-2 functions as a Tie2 agonist in tumor models, where it limits the effects of VEGF inhibition. Cancer Res..

[171] Hu B., Guo P., Fang Q., Tao H.Q., Wang D., Nagane M., Huang H.J.S., Gunji Y., Nishikawa R., Alitalo K. (2003). Angiopoietin-2 induces human glioma invasion through the activation of matrix metalloprotease-2. Proc. Natl. Acad. Sci. USA.

[172] Hu B., Jarzynka M.J., Guo P., Imanishi Y., Schlaepfer D.D., Cheng S.Y. (2006). Angiopoietin 2 induces glioma cell invasion by stimulating matrix metalloprotease 2 expression through the αvβ1 integrin and focal adhesion kinase signaling pathway. Cancer Res..

[173] Coffelt S.B., Tal A.O., Scholz A., De Palma M., Patel S., Urbich C., Biswas S.K., Murdoch C., Plate K.H., Reiss Y. (2010). Angiopoietin-2 regulates gene expression in TIE2-expressing monocytes and augments their inherent proangiogenic functions. Cancer Res..

[174] Gabrusiewicz K., Liu D., Cortes-Santiago N., Hossain M.B., Conrad C.A., Aldape K.D., Fuller G.N., Marini F.C., Alonso M.M., Idoate M.A. (2014). Anti-vascular endothelial growth factor therapy-induced glioma invasion is associated with accumulation of Tie2-expressing monocytes. Oncotarget.

[175] Cortes-Santiago N., Hossain M.B., Gabrusiewicz K., Fan X., Gumin J., Marini F.C., Alonso M.M., Lang F., Yung W.K., Fueyo J. (2016). Soluble Tie2 overrides the heightened invasion induced by anti-angiogenesis therapies in gliomas. Oncotarget.

[176] Carafoli F., Hohenester E. (2013). Collagen recognition and transmembrane signalling by discoidin domain receptors. Biochim. Biophys. Acta Proteins Proteom..

[177] Vogel W., Gish G.D., Alves F., Pawson T. (1997). The Discoidin Domain Receptor Tyrosine Kinases Are Activated by Collagen. Mol. Cell.

[178] Valiathan R.R., Marco M., Leitinger B., Kleer C.G., Fridman R. (2012). Discoidin domain receptor tyrosine kinases: New players in cancer progression. Cancer Metastasis Rev..

[179] Payne L.S., Huang P.H. (2013). The pathobiology of collagens in glioma. Mol. Cancer Res..

[180] Weiner H.L., Rothman M., Miller D.C., Ziff E.B. (1996). Pediatric brain tumors express multiple receptor tyrosine kinases including novel cell adhesion kinases. Pediatr. Neurosurg..

[181] Weiner H.L., Huang H., Zagzag D., Boyce H., Lichtenbaum R., Ziff E.B. (2000). Consistent and Selective Expression of the Discoidin Domain Receptor-1 Tyrosine Kinase in Human Brain Tumors. Neurosurgery.

[182] Ram R., Lorente G., Nikolich K., Urfer R., Foehr E., Nagavarapu U. (2006). Discoidin Domain Receptor-1a (DDR1a) Promotes Glioma Cell Invasion and Adhesion in Association with Matrix Metalloproteinase-2. J. Neurooncol..

[183] Yamanaka R., Arao T., Yajima N., Tsuchiya N., Homma J., Tanaka R., Sano M., Oide A., Sekijima M., Nishio K. (2006). Identification of expressed genes characterizing long-term survival in malignant glioma patients. Oncogene.

[184] El Husseini K., Marguet F., Lamy A., Magne N., Fontanilles M. (2020). Major response to temozolomide as first-line treatment for newly-diagnosed DDR2-mutated glioblastoma: A case report. Rev. Neurol..

[185] Arighi E., Borrello M.G., Sariola H. (2005). RET tyrosine kinase signaling in development and cancer. Cytokine Growth Factor Rev..

[186] Pachnis V., Mankoo B., Costantini F. (1993). Expression of the c-ret proto-oncogene during mouse embryogenesis. Development.

[187] Bonanomi D., Chivatakarn O., Bai G., Abdesselem H., Lettieri K., Marquardt T., Pierchala B.A., Pfaff S.L. (2012). Ret Is a Multifunctional Coreceptor that Integrates Diffusible- and Contact-Axon Guidance Signals. Cell.

[188] Pierchala B.A. (2006). Glial Cell Line-Derived Neurotrophic Factor-Dependent Recruitment of Ret into Lipid Rafts Enhances Signaling by Partitioning Ret from Proteasome-Dependent Degradation. J. Neurosci..

[189] Wiesenhofer B., Stockhammer G., Kostron H., Maier H., Hinterhuber H., Humpel C. (2000). Glial cell line-derived neurotrophic factor (GDNF) and its receptor (GFR-α1) are strongly expressed in human gliomas. Acta Neuropathol..

[190] Kato S., Subbiah V., Marchlik E., Elkin S.K., Carter J.L., Kurzrock R. (2017). RET Aberrations in Diverse Cancers: Next-Generation Sequencing of 4,871 Patients. Clin. Cancer Res..

[191] Birchmeier C., O’Neill K., Riggs M., Wigler M. (1990). Characterization of ROS1 cDNA from a human glioblastoma cell line. Proc. Natl. Acad. Sci. USA.

[192] El-Deeb I.M., Yoo K.H., Lee S.H. (2010). ROS receptor tyrosine kinase: A new potential target for anticancer drugs. Med. Res. Rev..

[193] Birchmeier C., Sharma S., Wigler M. (1987). Expression and rearrangement of the ROS1 gene in human glioblastoma cells. Proc. Natl. Acad. Sci. USA.

[194] Jun H.J., Woolfenden S., Coven S., Lane K., Bronson R., Housman D., Charest A. (2009). Epigenetic Regulation of c-ROS Receptor Tyrosine Kinase Expression in Malignant Gliomas. Cancer Res..

[195] Pawson T. (2007). Dynamic control of signaling by modular adaptor proteins. Curr. Opin. Cell Biol..

[196] Batistatou A., Zioga A., Panelos J., Massi D., Agnantis N.J., Charalabopoulos K. (2007). A new concept of melanocytic neoplasia pathogenesis based on the phenotype of common acquired nevi. Med. Hypotheses.

[197] Hall B.E., Bar-Sagi D., Nassar N. (2002). The structural basis for the transition from Ras-GTP to Ras-GDP. Proc. Natl. Acad. Sci. USA.

[198] Rajasekhar V.K., Viale A., Socci N.D., Wiedmann M., Hu X., Holland E.C. (2003). Oncogenic Ras and Akt signaling contribute to glioblastoma formation by differential recruitment of existing mRNAs to polysomes. Mol. Cell.

[199] Cheng C.K., Fan Q.W., Weiss W.A. (2009). PI3K signaling in glioma—Animal models and therapeutic challenges. Brain Pathol..

[200] Kim I., Kim H.G., Moon S.O., Chae S.W., So J.N., Koh K.N., Ahn B.C., Koh G.Y. (2000). Angiopoietin-1 induces endothelial cell sprouting through the activation of focal adhesion kinase and plasmin secretion. Circ. Res..

[201] Kim I., Kim J.H., Moon S.O., Kwak H.J., Kim N.G., Koh G.Y. (2000). Angiopoietin-2 at high concentration can enhance enthelial cell survival through the phosphatidylinositol 3′-kinase/Akt signal transduction pathway. Oncogene.

[202] Vivanco I., Sawyers C.L. (2002). The phosphatidylinositol 3-kinase-AKT pathway in humancancer. Nat. Rev. Cancer.

[203] Sunayama J., Matsuda K.I., Sato A., Tachibana K., Suzuki K., Narita Y., Shibui S., Sakurada K., Kayama T., Tomiyama A. (2010). Crosstalk between the PI3K/mTOR and MEK/ERK pathways involved in the maintenance of self-renewal and tumorigenicity of glioblastoma stem-like cells. Stem Cells.

[204] Mendoza M.C., Er E.E., Blenis J. (2011). The Ras-ERK and PI3K-mTOR pathways: Cross-talk and compensation. Trends Biochem. Sci..

[205] Jeuken J., van de Broecke C., Gijsen S., Boots-Sprenger S., Wesseling P. (2007). RAS/RAF pathway activation in gliomas: The result of copy number gains rather than activating mutations. Acta Neuropathol..

[206] Laplante M., Sabatini D.M. (2009). mTOR signaling at a glance. J. Cell Sci..

[207] Sen B., Xie Z., Case N., Thompson W.R., Uzer G., Styner M., Rubin J. (2014). MTORC2 regulates mechanically induced cytoskeletal reorganization and lineage selection in marrow-derived mesenchymal stem cells. J. Bone Miner. Res..

[208] Mecca C., Giambanco I., Donato R., Arcuri C. (2018). Targeting mTOR in glioblastoma: Rationale and preclinical/clinical evidence. Dis. Markers.

[209] Hauck C.R., Hsia D.A., Schlaepfer D.D. (2002). The focal adhesion kinase—A regulator of cell migration and invasion. IUBMB Life.

[210] Guo W., Giancotti F.G. (2004). Integrin signalling during tumour progression. Nat. Rev. Mol. Cell Biol..

[211] Hu J., Mukhopadhyay A., Truesdell P., Chander H., Mukhopadhyay U.K., Mak A.S., Craig A.W.B. (2011). Cdc42-interacting protein 4 is a Src substrate that regulates invadopodia and invasiveness of breast tumors by promoting MT1-MMP endocytosis. J. Cell Sci..

[212] Hecker T.P., Grammer J.R., Gillespie G.Y., Stewart J., Gladson C.L. (2002). Focal adhesion kinase enhances signaling through the Shc/extracellular signal-regulated kinase pathway in anaplastic astrocytoma tumor biopsy samples. Cancer Res..

[213] Golubovskaya V.M., Huang G., Ho B., Yemma M., Morrison C.D., Lee J., Eliceiri B.P., Cance W.G. (2013). Pharmacologic blockade of FAK autophosphorylation decreases human glioblastoma tumor growth and synergizes with temozolomide. Mol. Cancer Ther..

[214] Wills M.K.B., Jones N. (2012). Teaching an old dogma new tricks: Twenty years of Shc adaptor signalling. Biochem. J..

[215] Wills M.K.B., Chahi A.K., Lau H.R., Tilak M., Guild B.D., New L.A., Lu P., Jacquet K., Meakin S.O., Bisson N. (2017). Signaling adaptor ShcD suppresses extracellular signal-regulated kinase (Erk) phosphorylation distal to the Ret and Trk neurotrophic receptors. J. Biol. Chem..

[216] Audero E., Cascone I., Maniero F., Napione L., Arese M., Lanfrancone L., Bussolino F. (2004). Adaptor ShcA Protein Binds Tyrosine Kinase Tie2 Receptor and Regulates Migration and Sprouting but Not Survival of Endothelial Cells. J. Biol. Chem..

[217] Jones N., Master Z., Jones J., Bouchard D., Gunji Y., Sasaki H., Daly R., Alitalo K., Dumont D.J. (1999). Identification of Tek/Tie2 binding partners. Binding to a multifunctional docking site mediates cell survival and migration. J. Biol. Chem..

[218] Mayer B.J. (2012). Perspective: Dynamics of receptor tyrosine kinase signaling complexes. FEBS Lett..

[219] Jadwin J.A., Curran T.G., Lafontaine A.T., White F.M., Mayer B.J. (2018). Src homology 2 domains enhance tyrosine phosphorylation in vivo by protecting binding sites in their target proteins from dephosphorylation. J. Biol. Chem..

[220] Wills M.K.B., Tong J., Tremblay S.L., Moran M.F., Jones N. (2014). The ShcD signaling adaptor facilitates ligand-independent phosphorylation of the EGF receptor. Mol. Biol. Cell.

[221] Tilak M., Alural B., Wismer S.E., Brasher M.I., New L.A., Sheridan S.D., Perlis R.H., Coppolino M.G., Lalonde J., Jones N. (2021). Adaptor protein ShcD interacts with Tie2 receptor to synergistically promote glioma cell invasion. Mol. Cancer Res..

[222] Weis W.I., Kobilka B.K. (2018). The Molecular Basis of G Protein-Coupled Receptor Activation. Annu. Rev. Biochem..

[223] Daub H., Weiss F.U., Wallasch C., Ullrich A. (1996). Role of transactivation of the EGF receptor in signalling by G-protein-coupled receptors. Nature.

[224] Cattaneo F., Guerra G., Parisi M., De Marinis M., Tafuri D., Cinelli M., Ammendola R. (2014). Cell-surface receptors transactivation mediated by G protein-coupled receptors. Int. J. Mol. Sci..

[225] Prenzel N., Zwick E., Daub H., Leserer M., Abraham R., Wallasch C., Ullrich A. (1999). EGF receptor transactivation by G-protein-coupled receptors requires metalloproteinase cleavage of proHB-EGF. Nature.

[226] Sternlicht M.D., Werb Z. (2001). How matrix metalloproteinases regulate cell behavior. Annu. Rev. Cell Dev. Biol..

[227] Borroto-Escuela D.O., Romero-Fernandez W., Mudó G., Pérez-Alea M., Ciruela F., Tarakanov A.O., Narvaez M., Di Liberto V., Agnati L.F., Belluardo N. (2012). Fibroblast growth factor receptor 1 5-hydroxytryptamine 1A heteroreceptor complexes and their enhancement of hippocampal plasticity. Biol. Psychiatry.

[228] El-Shewy H.M., Abdel-Samie S.A., al Qalam A.M., Lee M.H., Kitatani K., Anelli V., Jaffa A.A., Obeid L.M., Luttrell L.M. (2011). Phospholipase C and protein kinase C-β 2 mediate insulin-like growth factor ii-dependent sphingosine kinase 1 activation. Mol. Endocrinol..

[229] Chen J.-K., Chen J.-K., Harris R.C. (2012). Angiotensin II Induces Epithelial-to-Mesenchymal Transition in Renal Epithelial Cells through Reactive Oxygen Species/Src/Caveolin-Mediated Activation of an Epidermal Growth Factor Receptor-Extracellular Signal-Regulated Kinase Signaling Pathway. Mol. Cell. Biol..

[230] Belcheva M.M., Haas P.D., Tan Y., Heaton V.M., Coscia C.J. (2002). The fibroblast growth factor receptor is at the site of convergence between μ-opioid receptor and growth factor signaling pathways in rat C6 glioma cells. J. Pharmacol. Exp. Ther..

[231] Huang J., Hu J., Bian X., Chen K., Gong W., Dunlop N.M., Howard O.M.Z., Ji M.W. (2007). Transactivation of the epidermal growth factor receptor by formylpeptide receptor exacerbates the malignant behavior of human glioblastoma cells. Cancer Res..

[232] Sciaccaluga M., D’Alessandro G., Pagani F., Ferrara G., Lopez N., Warr T., Gorello P., Porzia A., Mainiero F., Santoro A. (2013). Functional cross talk between CXCR4 and PDGFR on glioblastoma cells is essential for migration. PLoS ONE.

[233] Delcourt N., Bockaert J., Marin P. (2007). GPCR-jacking: From a new route in RTK signalling to a new concept in GPCR activation. Trends Pharmacol. Sci..

[234] Clapham D.E. (2007). Calcium Signaling. Cell.

[235] Leclerc C., Haeich J., Aulestia F.J., Kilhoffer M.C., Miller A.L., Néant I., Webb S.E., Schaeffer E., Junier M.P., Chneiweiss H. (2016). Calcium signaling orchestrates glioblastoma development: Facts and conjunctures. Biochim. Biophys. Acta Mol. Cell Res..

[236] Maklad A., Sharma A., Azimi I. (2019). Calcium signaling in brain cancers: Roles and therapeutic targeting. Cancers.

[237] Kang S.S., Han K.S., Ku B.M., Lee Y.K., Hong J., Shin H.Y., Almonte A.G., Woo D.H., Brat D.J., Hwang E.M. (2010). Caffeine-mediated inhibition of calcium release channel inositol 1,4,5-trisphosphate receptor subtype 3 blocks glioblastoma invasion and extends survival. Cancer Res..

[238] Kang S., Hong J., Lee J.M., Moon H.E., Jeon B., Choi J., Yoon N.A., Paek S.H., Roh E.J., Lee C.J. (2017). Trifluoperazine, a well-known antipsychotic, inhibits glioblastoma invasion by binding to calmodulin and disinhibiting calcium release channel IP3R. Mol. Cancer Ther..

[239] Mahalingam D., Wilding G., Denmeade S., Sarantopoulas J., Cosgrove D., Cetnar J., Azad N., Bruce J., Kurman M., Allgood V.E. (2016). Mipsagargin, a novel thapsigargin-based PSMA-Activated prodrug: Results of a first-in-man phase i clinical trial in patients with refractory, advanced or metastatic solid tumours. Br. J. Cancer.

[240] Mahalingam D., Peguero J., Cen P., Arora S.P., Sarantopoulos J., Rowe J., Allgood V., Tubb B., Campos L. (2019). A phase II, multicenter, single-arm study of mipsagargin (G-202) as a second-line therapy following sorafenib for adult patients with progressive advanced hepatocellular carcinoma. Cancers.

[241] Prakriya M., Lewis R.S. (2015). Store-operated calcium channels. Physiol. Rev..

[242] Xie J., Pan H., Yao J., Zhou Y., Han W. (2016). SOCE and cancer: Recent progress and new perspectives. Int. J. Cancer.

[243] Chen Y.F., Lin P.C., Yeh Y.M., Chen L.H., Shen M.R. (2019). Store-Operated Ca2+ entry in tumor progression: From molecular mechanisms to clinical implications. Cancers.

[244] Motiani R.K., Hyzinski-García M.C., Zhang X., Henkel M.M., Abdullaev I.F., Kuo Y.H., Matrougui K., Mongin A.A., Trebak M. (2013). STIM1 and Orai1 mediate CRAC channel activity and are essential for human glioblastoma invasion. Pflug. Arch. Eur. J. Physiol..

[245] Zhu M., Chen L., Zhao P., Zhou H., Zhang C., Yu S., Lin Y., Yang X. (2014). Store-operated Ca2+ entry regulates glioma cell migration and invasion via modulation of Pyk2 phosphorylation. J. Exp. Clin. Cancer Res..

[246] Yuan F., Yi L., Hai L., Wang Y., Yang Y., Li T., Tong L., Ma H., Liu P., Ming H. (2019). Identification of Key Pathways and Genes in the Orai2 Mediated Classical and Mesenchymal Subtype of Glioblastoma by Bioinformatic Analyses. Dis. Markers.

[247] Shen T., Guo Q. (2018). Role of Pyk2 in human cancers. Med. Sci. Monit..

[248] Emeriau N., de Clippele M., Gailly P., Tajeddine N. (2018). Store operated calcium entry is altered by the inhibition of receptors tyrosine kinase. Oncotarget.

[249] Van Tellingen O., Yetkin-Arik B., De Gooijer M.C., Wesseling P., Wurdinger T., De Vries H.E. (2015). Overcoming the blood-brain tumor barrier for effective glioblastoma treatment. Drug Resist. Updates.

[250] Talasila K.M., Soentgerath A., Euskirchen P., Rosland G.V., Wang J., Huszthy P.C., Prestegarden L., Skaftnesmo K.O., Sakariassen P.Ø., Eskilsson E. (2013). EGFR wild-type amplification and activation promote invasion and development of glioblastoma independent of angiogenesis. Acta Neuropathol..

[251] Orellana L. (2019). Convergence of EGFR glioblastoma mutations: Evolution and allostery rationalizing targeted therapy. Mol. Cell. Oncol..

[252] Li J., Liang R., Song C., Xiang Y., Liu Y. (2018). Prognostic significance of epidermal growth factor receptor expression in glioma patients. Onco. Targets Ther..

[253] Krawczyk P., Kowalski D.M., Ramlau R., Kalinka-Warzocha E., Winiarczyk K., Stencel K., Powrózek T., Reszka K., Wojas-Krawczyk K., Bryl M. (2017). Comparison of the effectiveness of erlotinib, gefitinib, and afatinib for treatment of non-small cell lung cancer in patients with common and rare EGFR gene mutations. Oncol. Lett..

[254] Prados M.D., Lamborn K.R., Chang S., Burton E., Butowski N., Malec M., Kapadia A., Rabbit J., Page M.S., Fedoroff A. (2006). Phase 1 study of erlotinib HCl alone and combined with temozolomide in patients with stable or recurrent malignant glioma. Neuro. Oncol..

[255] Halatsch M.E., Gehrke E.E., Vougioukas V.I., Bötefür I.C., Borhani F.A., Efferth T., Gebhart E., Domhof S., Schmidt U., Buchfelder M. (2004). Inverse correlation of epidermal growth factor receptor messenger RNA induction and suppression of anchorage-independent growth by OSI-774, an epidermal growth factor receptor tyrosine kinase inhibitor, in glioblastoma multiforme cell lines. J. Neurosurg..

[256] Griffero F., Daga A., Marubbi D., Capra M.C., Melotti A., Pattarozzi A., Gatti M., Bajetto A., Porcile C., Barbieri F. (2009). Different response of human glioma tumor-initiating cells to epidermal growth factor receptor kinase inhibitors. J. Biol. Chem..

[257] Van Den Bent M.J., Brandes A.A., Rampling R., Kouwenhoven M.C.M., Kros J.M., Carpentier A.F., Clement P.M., Frenay M., Campone M., Baurain J.F. (2009). Randomized phase II trial of erlotinib versus temozolomide or carmustine in recurrent glioblastoma: EORTC brain tumor group study 26034. J. Clin. Oncol..

[258] Peereboom D.M., Shepard D.R., Ahluwalia M.S., Brewer C.J., Agarwal N., Stevens G.H.J., Suh J.H., Toms S.A., Vogelbaum M.A., Weil R.J. (2010). Phase II trial of erlotinib with temozolomide and radiation in patients with newly diagnosed glioblastoma multiforme. J. Neurooncol..

[259] Arif S., Pandith A., Tabasum R., Ramzan A., Singh S., Siddiqi M., Bhat A. (2018). Significant effect of anti-tyrosine kinase inhibitor (Gefitinib) on overall survival of the glioblastoma multiforme patients in the backdrop of mutational status of epidermal growth factor receptor and PTEN Genes. Asian J. Neurosurg..

[260] Bethune G., Bethune D., Ridgway N., Xu Z. (2010). Epidermal growth factor receptor (EGFR) in lung cancer: An overview and update. J. Thorac. Dis..

[261] Binder Z.A., Thorne A.H., Bakas S., Wileyto E.P., Bilello M., Akbari H., Rathore S., Ha S.M., Zhang L., Ferguson C.J. (2018). Epidermal Growth Factor Receptor Extracellular Domain Mutations in Glioblastoma Present Opportunities for Clinical Imaging and Therapeutic Development. Cancer Cell.

[262] Vivanco I., Ian Robins H., Rohle D., Campos C., Grommes C., Nghiemphu P.L., Kubek S., Oldrini B., Chheda M.G., Yannuzzi N. (2012). Differential sensitivity of glioma- versus lung cancer-specific EGFR mutations to EGFR kinase inhibitors. Cancer Discov..

[263] Reardon D.A., Nabors L.B., Mason W.P., Perry J.R., Shapiro W., Kavan P., Mathieu D., Phuphanich S., Cseh A., Fu Y. (2015). Phase I/randomized phase II study of afatinib, an irreversible ErbB family blocker, with or without protracted temozolomide in adults with recurrent glioblastoma. Neuro. Oncol..

[264] Vengoji R., Macha M.A., Nimmakayala R.K., Rachagani S., Siddiqui J.A., Mallya K., Gorantla S., Jain M., Ponnusamy M.P., Batra S.K. (2019). Afatinib and Temozolomide combination inhibits tumorigenesis by targeting EGFRvIII-cMet signaling in glioblastoma cells. J. Exp. Clin. Cancer Res..

[265] Liu X., Chen X., Shi L., Shan Q., Cao Q., Yue C., Li H., Li S., Wang J., Gao S. (2019). The third-generation EGFR inhibitor AZD9291 overcomes primary resistance by continuously blocking ERK signaling in glioblastoma. J. Exp. Clin. Cancer Res..

[266] Elsamadicy A.A., Chongsathidkiet P., Desai R., Woroniecka K., Farber S.H., Fecci P.E., Sampson J.H. (2017). Prospect of rindopepimut in the treatment of glioblastoma. Expert Opin. Biol. Ther..

[267] Carboni J.M., Wittman M., Yang Z., Lee F., Greer A., Hurlburt W., Hillerman S., Cao C., Cantor G.H., Dell-John J. (2009). BMS-754807, a small molecule inhibitor of insulin-like growth factor-1R/IR. Mol. Cancer Ther..

[268] Schwartz G.K., Dickson M.A., Lorusso P.M., Sausville E.A., Maekawa Y., Watanabe Y., Kashima N., Nakashima D., Akinaga S. (2016). Preclinical and first-in-human phase I studies of KW-2450, An oral tyrosine kinase inhibitor with insulin-like growth factor receptor-1/insulin receptor selectivity. Cancer Sci..

[269] Simpson A., Petnga W., Macaulay V.M., Weyer-Czernilofsky U., Bogenrieder T. (2017). Insulin-Like Growth Factor (IGF) Pathway Targeting in Cancer: Role of the IGF Axis and Opportunities for Future Combination Studies. Target. Oncol..

[270] Yin S., Girnita A., Strömberg T., Khan Z., Andersson S., Zheng H., Ericsson C., Axelson M., Nistér M., Larsson O. (2010). Targeting the insulin-like growth factor-1 receptor by picropodophyllin as a treatment option for glioblastoma. Neuro. Oncol..

[271] Iqbal N., Iqbal N. (2014). Imatinib: A Breakthrough of Targeted Therapy in Cancer. Chemother. Res. Pract..

[272] Raymond E., Brandes A.A., Dittrich C., Fumoleau P., Coudert B., Clement P.M.J., Frenay M., Rampling R., Stupp R., Kros J.M. (2008). Phase II study of imatinib in patients with recurrent gliomas of various histologies: A European organisation for research and treatment of cancer brain tumor group study. J. Clin. Oncol..

[273] Wen P.Y., Yung W.K.A., Lamborn K.R., Dahia P.L., Wang Y., Peng B., Abrey L.E., Raizer J., Cloughesy T.F., Fink K. (2006). Phase I/II study of imatinib mesylate for recurrent malignant gliomas: North American Brain Tumor Consortium Study 99-08. Clin. Cancer Res..

[274] Frolov A., Evans I.M., Li N., Sidlauskas K., Paliashvili K., Lockwood N., Barrett A., Brandner S., Zachary I.C., Frankel P. (2016). Imatinib and Nilotinib increase glioblastoma cell invasion via Abl-independent stimulation of p130Cas and FAK signalling. Sci. Rep..

[275] Oertel S., Krempien R., Lindel K., Zabel A., Milker-Zabel S., Bischof M., Lipson K.E., Peschke P., Debus J., Abdollahi A. (2006). Human glioblastoma and carcinoma xenograft tumors treated by combined radiation and imatinib (Gleevec^®^). Strahlenther. Onkol..

[276] Odia Y., Sul J., Shih J.H., Kreisl T.N., Butman J.A., Iwamoto F.M., Fine H.A. (2016). A Phase II trial of tandutinib (MLN 518) in combination with bevacizumab for patients with recurrent glioblastoma. CNS Oncol..

[277] Griswold I.J., Shen L.J., La Rosée P., Demehri S., Heinrich M.C., Braziel R.M., McGreevey L., Haley A.D., Giese N., Druker B.J. (2004). Effects of MLN518, a dual FLT3 and KTT inhibitor, on normal and malignant hematopoiesis. Blood.

[278] Ferrara N., Hillan K.J., Gerber H.P., Novotny W. (2004). Discovery and development of bevacizumab, an anti-VEGF antibody for treating cancer. Nat. Rev. Drug Discov..

[279] Hu-Lowe D.D., Zou H.Y., Grazzini M.L., Hallin M.E., Wickman G.R., Amundson K., Chen J.H., Rewolinski D.A., Yamazaki S., Wu E.Y. (2008). Nonclinical antiangiogenesis and antitumor activities of axitinib (AG-013736), an oral, potent, and selective inhibitor of vascular endothelial growth factor receptor tyrosine kinases 1, 2, 3. Clin. Cancer Res..

[280] Duerinck J., Du Four S., Vandervorst F., D’Haene N., Le Mercier M., Michotte A., Van Binst A.M., Everaert H., Salmon I., Bouttens F. (2016). Randomized phase II study of axitinib versus physicians best alternative choice of therapy in patients with recurrent glioblastoma. J. Neurooncol..

[281] Siegelin M.D., Raskett C.M., Gilbert C.A., Ross A.H., Altieri D.C. (2010). Sorafenib exerts anti-glioma activity in vitro and in vivo. Neurosci. Lett..

[282] Mendel D.B., Douglas Laird A., Xin X., Louie S.G., Christensen J.G., Li G., Schreck R.E., Abrams T.J., Ngai T.J., Lee L.B. (2003). In vivo antitumor activity of SU11248, a novel tyrosine kinase inhibitor targeting vascular endothelial growth factor and platelet-derived growth factor receptors: Determination of a pharmacokinetic/pharmacodynamic relationship. Clin. Cancer Res..

[283] Hottinger A.F., Aissa A.B., Espeli V., Squiban D., Dunkel N., Vargas M.I., Hundsberger T., MacH N., Schaller K., Weber D.C. (2014). Phase i study of sorafenib combined with radiation therapy and temozolomide as first-line treatment of high-grade glioma. Br. J. Cancer.

[284] Neyns B., Sadones J., Chaskis C., Dujardin M., Everaert H., Lv S., Duerinck J., Tynninen O., Nupponen N., Michotte A. (2011). Phase II study of sunitinib malate in patients with recurrent high-grade glioma. J. Neurooncol..

[285] Katoh M. (2016). FGFR inhibitors: Effects on cancer cells, tumor microenvironment and whole-body homeostasis (Review). Int. J. Mol. Med..

[286] Lassman A., Sepúlveda-Sánchez J., Cloughesy T., Gil-Gil J., Puduvalli V., Raizer J., De Vos F., Wen P., Butowski N., Clement P. (2019). Actr-33. Infigratinib (Bgj398) in Patients With Recurrent Gliomas with Fibroblast Growth Factor Receptor (Fgfr) Alterations: A Multicenter Phase Ii Study. Neuro. Oncol..

[287] Wadhwa S., Nag T.C., Jindal A., Kushwaha R., Mahapatra A.K., Sarkar C. (2003). Expression of the neurotrophin receptors Trk A and Trk B in adult human astrocytoma and glioblastoma. J. Biosci..

[288] Assimakopoulou M., Kondyli M., Gatzounis G., Maraziotis T., Varakis J. (2007). Neurotrophin receptors expression and JNK pathway activation in human astrocytomas. BMC Cancer.

[289] Pinet S., Bessette B., Vedrenne N., Lacroix A., Richard L., Jauberteau M.O., Battu S., Lalloué F. (2016). TrkB-containing exosomes promote the transfer of glioblastoma aggressiveness to YKL-40-inactivated glioblastoma cells. Oncotarget.

[290] Cazorla M., Prémont J., Mann A., Girard N., Kellendonk C., Rognan D. (2011). Identification of a low-molecular weight TrkB antagonist with anxiolytic and antidepressant activity in mice. J. Clin. Investig..

[291] Ni J., Xie S., Ramkissoon S.H., Luu V., Sun Y., Bandopadhayay P., Beroukhim R., Roberts T.M., Stiles C.D., Segal R.A. (2017). Tyrosine receptor kinase B is a drug target in astrocytomas. Neuro. Oncol..

[292] Cocco E., Scaltriti M., Drilon A. (2018). NTRK fusion-positive cancers and TRK inhibitor therapy. Nat. Rev. Clin. Oncol..

[293] Xu T., Wang H., Huang X., Li W., Huang Q., Yan Y., Chen J. (2018). Gene Fusion in Malignant Glioma: An Emerging Target for Next-Generation Personalized Treatment. Transl. Oncol..

[294] Ardini E., Menichincheri M., Banfi P., Bosotti R., De Ponti C., Pulci R., Ballinari D., Ciomei M., Texido G., Degrassi A. (2016). Entrectinib, a Pan-TRK, ROS1, and ALK inhibitor with activity in multiple molecularly defined cancer indications. Mol. Cancer Ther..

[295] Doebele R.C., Davis L.E., Vaishnavi A., Le A.T., Estrada-Bernal A., Keysar S., Jimeno A., Varella-Garcia M., Aisner D.L., Li Y. (2015). An oncogenic NTRK fusion in a patient with soft-tissue sarcoma with response to the tropomyosin-related kinase inhibitor LOXO-101. Cancer Discov..

[296] Drilon A., Siena S., Ou S.H.I., Patel M., Ahn M.J., Lee J., Bauer T.M., Farago A.F., Wheler J.J., Liu S.V. (2017). Safety and antitumor activity of the multitargeted pan-TRK, ROS1, and ALK inhibitor entrectinib: Combined results from two phase I trials (ALKA-372-001 and STARTRK-1). Cancer Discov..

[297] Drilon A., Laetsch T.W., Kummar S., Dubois S.G., Lassen U.N., Demetri G.D., Nathenson M., Doebele R.C., Farago A.F., Pappo A.S. (2018). Efficacy of larotrectinib in TRK fusion-positive cancers in adults and children. N. Engl. J. Med..

[298] Laetsch T.W., DuBois S.G., Mascarenhas L., Turpin B., Federman N., Albert C.M., Nagasubramanian R., Davis J.L., Rudzinski E., Feraco A.M. (2018). Larotrectinib for paediatric solid tumours harbouring NTRK gene fusions: Phase 1 results from a multicentre, open-label, phase 1/2 study. Lancet Oncol..

[299] Drilon A., Nagasubramanian R., Blake J.F., Ku N., Tuch B.B., Ebata K., Smith S., Lauriault V., Kolakowski G.R., Brandhuber B.J. (2017). A next-generation TRK kinase inhibitor overcomes acquired resistance to prior trk kinase inhibition in patients with TRK fusion-positive solid tumors. Cancer Discov..

[300] Drilon A., Ou S.H.I., Cho B.C., Kim D.W., Lee J., Lin J.J., Zhu V.W., Ahn M.J., Camidge D.R., Nguyen J. (2018). Repotrectinib (Tpx-0005) is a next-generation ros1/trk/alk inhibitor that potently inhibits ros1/trk/alk solvent-front mutations. Cancer Discov..

[301] Park J.S., Kim I.K., Han S., Park I., Kim C., Bae J., Oh S.J., Lee S., Kim J.H., Woo D.C. (2016). Normalization of Tumor Vessels by Tie2 Activation and Ang2 Inhibition Enhances Drug Delivery and Produces a Favorable Tumor Microenvironment. Cancer Cell.

[302] Reusch P., Barleon B., Weindel K., Martiny-Baron G., Gödde A., Siemeister G., Marmé D. (2001). Identification of a soluble form of the angiopoietin receptor TIE-2 released from endothelial cells and present in human blood. Angiogenesis.

[303] Harney A.S., Karagiannis G.S., Pignatelli J., Smith B.D., Kadioglu E., Wise S.C., Hood M.M., Kaufman M.D., Leary C.B., Lu W.P. (2017). The selective Tie2 inhibitor rebastinib blocks recruitment and function of Tie2Hi macrophages in breast cancer and pancreatic neuroendocrine tumors. Mol. Cancer Ther..

[304] Cam M., Charan M., Welker A.M., Dravid P., Studebaker A.W., Leonard J.R., Pierson C.R., Nakano I., Beattie C.E., Hwang E.I. (2020). ΔNp73/ETS2 complex drives glioblastoma pathogenesis- targeting downstream mediators by rebastinib prolongs survival in preclinical models of glioblastoma. Neuro. Oncol..

[305] Schneider H., Szabo E., Machado R.A.C., Broggini-Tenzer A., Walter A., Lobell M., Heldmann D., Süssmeier F., Grünewald S., Weller M. (2017). Novel TIE-2 inhibitor BAY-826 displays in vivo efficacy in experimental syngeneic murine glioma models. J. Neurochem..

[306] Piao Y., Park S.Y., Henry V., Smith B.D., Tiao N., Flynn D.L., De Groot J.F. (2016). Novel MET/TIE2/VEGFR2 inhibitor altiratinib inhibits tumor growth and invasiveness in bevacizumab-resistant glioblastoma mouse models. Neuro. Oncol..

[307] Pearson J.R.D., Regad T. (2017). Targeting cellular pathways in glioblastoma multiforme. Signal Transduct. Target. Ther..

[308] Monje M., Borniger J.C., D’Silva N.J., Deneen B., Dirks P.B., Fattahi F., Frenette P.S., Garzia L., Gutmann D.H., Hanahan D. (2020). Roadmap for the Emerging Field of Cancer Neuroscience. Cell.

[309] Venkatesh H.S., Johung T.B., Caretti V., Noll A., Tang Y., Nagaraja S., Gibson E.M., Mount C.W., Polepalli J., Mitra S.S. (2019). Neuronal activity promotes glioma growth through neuroligin-3 secretion. Cell Rep..

[310] Venkatesh H.S., Morishita W., Geraghty A.C., Silverbush D., Gillespie S.M., Arzt M., Tam L.T., Espenel C., Ponnuswami A., Ni L. (2019). Electrical and synaptic integration of glioma into neural circuits. Nature.

[311] Venkataramani V., Tanev D.I., Strahle C., Studier-Fischer A., Fankhauser L., Kessler T., Körber C., Kardorff M., Ratliff M., Xie R. (2019). Glutamatergic synaptic input to glioma cells drives brain tumour progression. Nature.

[312] Zeng Q., Michael I.P., Zhang P., Saghafinia S., Knott G., Jiao W., McCabe B.D., Galván J.A., Robinson H.P.C., Zlobec I. (2019). Synaptic proximity enables NMDAR signalling to promote brain metastasis. Nature.

[313] Tuveson D., Clevers H. (2019). Cancer modeling meets human organoid technology. Science.

[314] Linkous A., Balamatsias D., Snuderl M., Edwards L., Miyaguchi K., Milner T., Reich B., Cohen-Gould L., Storaska A., Nakayama Y. (2019). Modeling Patient-Derived Glioblastoma with Cerebral Organoids. Cell Rep..

[315] Ogawa J., Pao G.M., Shokhirev M.N., Verma I.M. (2018). Glioblastoma Model Using Human Cerebral Organoids. Cell Rep..

